# GWAS by Subtraction to Disentangle RBD Genetic Background from α-Synucleinopathies

**DOI:** 10.3390/ijms26083578

**Published:** 2025-04-10

**Authors:** Andrea Gaudio, Fabio Gotta, Clarissa Ponti, Alessandro Geroldi, Andrea La Barbera, Paola Mandich

**Affiliations:** 1 IRCCS Ospedale Policlinico San Martino–UOC Genetica Medica, Largo R. Benzi 10, 16132 Genova, Italy; fabio.gotta@hsanmartino.it (F.G.); andrea.labarbera@hsanmartino.it (A.L.B.); paola.mandich@unige.it (P.M.); 2 Department of Neuroscience, Rehabilitation, Ophthalmology, Genetics and Maternal and Child Health, University of Genova, Largo P. Daneo 3, 16132 Genova, Italy; clarissa.ponti@edu.unige.it (C.P.); alessandro.geroldi@unige.it (A.G.)

**Keywords:** RBD, GenomicSEM, GWAS-by-subtraction, α-synucleinopathies, Mendelian Randomization, *SNCA*, omics

## Abstract

Rapid eye movement (REM) sleep behavior disorder (RBD) is a parasomnia characterized by loss of muscle atonia and abnormal behaviors occurring during REM sleep. Idiopathic RBD (iRBD) is recognized as the strongest prodromal hallmark of α-synucleinopathies, with an established conversion rate to a neurodegenerative condition that reaches up to 96.6% at 15 years of follow-up. Moreover, RBD-converters display a more severe clinical trajectory compared to those that do not present with RBD. However, the extent to which iRBD represents a distinct genetic entity or an early manifestation of neurodegeneration remains unclear. To address this, we applied Genomic Structural Equation Modeling (GenomicSEM) using a GWAS-by-subtraction approach to disentangle the genetic architecture of iRBD from the shared genomic liability across α-synucleinopathies. Our findings highlight the *SNCA* locus as a key genetic regulator of iRBD susceptibility. While iRBD exhibits a partially distinct genetic signature, residual genomic overlap with neurodegenerative traits suggests that its genetic architecture exists along a continuum of α-synucleinopathy risk. In this scenario, the associations with neuroanatomical correlates may serve as early indicators of a trajectory toward future neurodegeneration. These findings provide a framework for identifying biomarkers that could aid in disease stratification and risk prediction, potentially improving early intervention strategies.

## 1. Introduction

Rapid eye movement (REM) sleep behavior disorder (RBD) is a parasomnia characterized by loss of muscle atonia and abnormal behaviors occurring during REM sleep [1]. RBD is described as either isolated RBD (iRBD) or in conjunction with neurological diseases, especially α-synucleinopathies such as Parkinson Disease (PD), Dementia with Lewy bodies (DLB), and Multiple System Atrophy (MSA) [1]. The onset of RBD often precedes neurodegeneration by several years [2]. The average conversion rate from RBD to a neurodegenerative disease is approximately 31.95% after a mean follow-up of 4.75 years, rising to 82.4% at 10.5 years, and reaching 96.6% at 14 years of follow-up [2]. Additionally, patients exhibiting RBD as one of the initial symptoms are at higher risk of rapid and severe cognitive decline [3], which makes RBD an interesting hallmark of neurodegeneration and a promising target for early diagnosis and intervention [4,5].

However, there is considerable variability in the timing and trajectory of this phenoconversion. Some individuals progress to PD, others to DLB or MSA, while others remain free of a diagnosed neurodegenerative disease for extended periods. This variability suggests that, while RBD is a robust predictor of neurodegeneration, it may also harbor signals specific to iRBD itself, which have yet to be fully elucidated [2].

By isolating RBD-specific signals, we can better understand the early molecular and genetic changes that precede full-blown neurodegeneration, and those independent from such processes.

To this end, we adapted the GWAS-by-subtraction strategy [6,7] to disentangle the genetic architecture of iRBD from its shared genomic risk associated to neurodegeneration. GenomicSEM [8,9] was leveraged to design a two-latent factor model that allowed us to partition the total genetic variance into two distinct components: one representing the residual genetic influences unique to iRBD, independent of neurodegeneration; the second capturing the overlapping genetic liability common to RBD, PD, DLB, and MSA, detached from iRBD specific signals.

We used results from the GWAS-by-Subtraction analysis to explore potentially pleiotropic loci [10] that could drive the trajectory of conversion from RBD to an α-synucleinopathy. Then, leveraging a set of highly heritable traits, we explored the genomic correlation of these traits with our latent factors [11]. Eventually, Mendelian Randomization (MR) [12] analysis allowed us to assess potential causal relationships with neuroanatomical changes that could differentiate intrinsic sleep disorder mechanisms from broader neurodegenerative processes.

## 2. Results

Linkage Disequilibrium Score Regression (LDSC) [11] analysis estimated single trait heritabilities and pairwise genetic correlation. All analyzed traits showed varying degrees of correlation (Supplementary Table S1). Notably, LDSC revealed a very strong out-of-scale genetic correlation between RBD and DLB, alongside a low heritability estimate for MSA (h^2^ Z ≈ 1). The high level of correlation between RBD and DLB likely reflects both the big difference in numerosity across the analyzed GWAS traits, as well as a tight biological link. Despite the low MSA heritability, we decided to include it due to biological assumptions.

**Figure 1:**
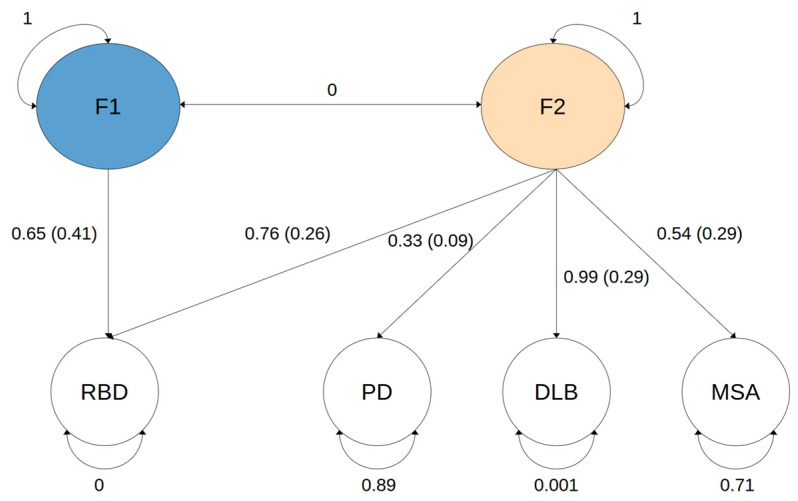
GenomicSEM graphical representation of the model. F1 and F2 represent latent, unobserved factors. Correlation between F1 and F2 set to 0. Latent factors variances set to 1. Numbers in straight arrows represent the factor loading for each trait.

To explore iRBD specific genomic associations, we used GenomicSEM [9] to design a model defined by two latent factors, F1 and F2 (Figure 1).

**Figure 2:**
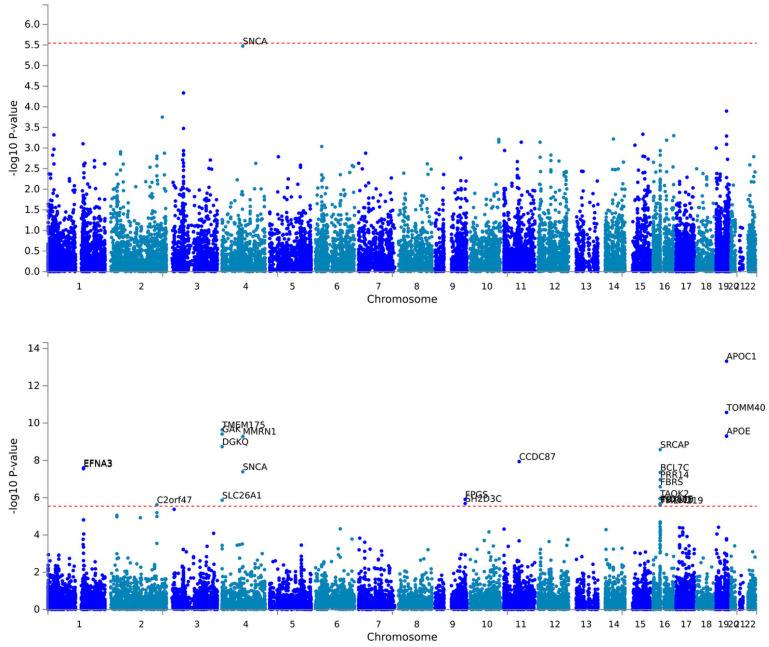
Top panel: F1 gene-based Manhattan plot. Bottom panel: F2 gene-based Manhattan plot. Red dashed line represents gene-based genome wide significance threshold set at 0.05/17,539 (Ensembl v85 number of protein coding genes).

To strongly isolate residual genetic variation in iRBD not explained by genetic variation in the genomic covariance between RBD, PD, DLB, and MSA, we superimposed absence of correlation between the two latent factors. RBD was regressed on latent factor F1, while all traits (RBD, PD, DLB, MSA) were regressed on latent factor F2, representing an mGWAS of shared genomic architecture between α-synucleinopathies without signals specific to iRBD. To ensure convergence in the model, we fixed certain constraints such as RBD, F1 and F2 residual variance set to zero, which are reflected as latent variables that are 100% heritable. The model fit indices suggested a good fit (CFI = 1; SRMR = 0.089). A graphical representation of the model is shown in Figure 1; detailed information available in Supplementary Table S1. Manhattan plots of gene-based tests for F1 and F2 are presented in Figure 2.

### 2.1. F1

#### 2.1.1. Genomic Risk Loci Definition, and Conditional and Joint Analysis

**Figure 3:**
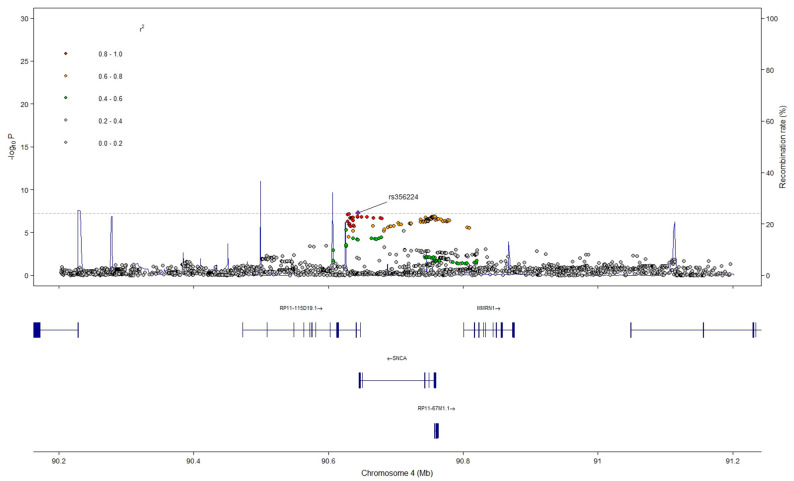
Locus zoom of F1 hit signal. Grey dashed line represents SNP-based genome wide significance threshold (*p* = 5 × 10^−8^). Created with https://cran.r-project.org/web//packages/locuszoomr/vignettes/locuszoomr.html (accessed on 10 February 2025; Version 0.3.8).

FUMA [13] settings for F1 were slightly refined due to the top hit signal’s *p* value being just below Bonferroni significance (*p* = 5.12 × 10^−8^). We set Bonferroni significance to 6 × 10^−8^, which allowed us to retain the top hit while avoiding the inclusion of non-significant spurious results. F1 GWAS identified a single genomic risk locus in chromosome 4 (Supplementary Table S1), with a single independent significant SNP, i.e., rs356224; 150 SNPs in LD that map to three genes: *SNCA*, *MMRN1*, *CCSER1*; and two anti-sense long non-coding RNA (lncRNA): RP11-115D19.1 and RP11-67M1.1 (*SNCA-AS1*) (Figure 3).

FINEMAP [14] analysis an estimated 83% posterior probability (PP) that the best credible set was explained by rs356224 and 99 SNPs in strong LD with it (median LD = 0.99), of which all exhibited small individual posterior probabilities (Supplementary Table S1). Further, we explored conditional and joint analysis leveraging GCTA-COJO [15]. Despite the GCTA team suggesting the use of a reference panel bigger than 1000 Genomes, the latter was used for ease of availability. Conditional analysis did not reveal any additional signals when accounting for the hit SNP effect. Joint analysis revealed a slightly decreased but still significant association (pJ = 6.9 × 10^−8^), with almost identical effect size (βJ = −0.76866) (Supplementary Table S1). This region contains SNPs in complete LD with the hit SNP, including SNPs whose association has been already identified in PD and RBD single-trait GWAS (e.g., rs356182) [16,17,18,19].

Rs356224 maps to RP11-115D19.1, a lncRNA that has been characterized in silico, in vitro and in vivo as a fine regulator of *SNCA* mRNA and protein expression, predominantly in brain regions, suggesting its role as a controller of α-synuclein levels [20,21].

#### 2.1.2. Colocalization

We leveraged colocalization analysis [22] to fine-map signals of F1 GWAS compared to F2, RBD, PD, DLB, and MSA using both the single causal variant assumption (coloc.abf) [23] and the multiple causal variants assumption (coloc.susie) [24]. MSA did not share variants in the selected region, so colocalization analysis was not performed. Colocalization with coloc.abf revealed a good separation of signals due to the designed model, with a low posterior probability for shared variants (PPH4) between F1 and F2 (PPH4 = 3.93%), as well as F1 and RBD (PPH4 = 1.25%) and F1 and DLB (PPH4 = 0.88%), with a higher PP4 between F1 and PD (PPH4 = 79.3%). When accounting for LD with multiple causal variants assumption, coloc.susie() highlighted a lower probability for causal variants to be shared between F1 and F2 (PPH4 = 0.24%), and a very low posterior probability for F1 and RBD (PPH4 = 0.04%), as well as F1 and DLB (PPH4 = 0.02%). Colocalization between F1 and PD used three SNP pairs, revealing a very low PPs in two out of three (PPH4 = 0.08%, PPH4 = 0.04%, respectively), with a slightly higher PP4 in the last SNP pair (PPH4 = 41%) (Supplementary Table S3).

#### 2.1.3. eQTL–sQTL Colocalization Analysis

We further inspected F1 GWAS results running a MetaXCan [25,26] analysis, both for eQTL and sQTL colocalization. eQTL analysis revealed a single strong Bonferroni significant signal in *APOE* gene within Substantia Nigra (Zmean = −1.82; *p* = 1.7 × 10^−8^); an additional three signals were identified just below the significance threshold in *SNCA* (Zmean = 1.94; *p* = 6.6 × 10^−6^), *GNAT1* (Zmean = 4.49; *p* = 7.27 × 10^−6^), and *SEMA3F* (Zmean = 4.38; *p* = 9.72 × 10^−6^) in the Cerebellar Hemisphere, Anterior Cingulate Cortex BA24, and Caudate basal ganglia, respectively (Supplementary Table S2). It should be noted that for the MetaXCan analysis, only 63% to 67% of available SNPs in the models were used. We attempted to leverage imputation within MetaXCan package, but saw no significant increase in this percentage, which remained between 64% and 67%.

### 2.2. F2

#### 2.2.1. Genomic Risk Loci Definition and Conditional and Joint Analysis

F2 mGWAS identified 97 genomic risk loci containing 117 independent significant SNPs mapped to 960 genes (Supplementary Table S1).

Conditional and joint analysis (COJO) suggested that the majority of significant SNPs, identified across chromosomes 1, 2, 4, 6, 8, 9, 10, 18, and 19, are likely the primary signals in their respective regions. However, the risk locus on chromosome 19 showed a more complex genetic architecture, with marked shifts in effect size and increased significance. This substantial change emphasizes the complex genetic architecture of this locus, likely driven by *APOE* regional LD structure, which is known to play a key role in neurodegenerative processes [27] (Supplementary Table S1).

#### 2.2.2. Colocalization

We inspected colocalization between F2 and F1, RBD, PD, and DLB using both the single causal variant assumption (coloc.abf) and the multiple causal variants assumption (coloc.susie) at locus chr4:90393623-90893623, selected due to its primary association with individual traits.

Colocalization with coloc.abf revealed a low posterior probability of sharing causal variants between F2 and RBD (PPH4 = 0.002%) and F2 and DLB (PPH4 = 4.29%), with a higher PP4 between F2 and PD (PPH4 = 97.1%). In the multiple causal variants assumption, coloc.susie() revealed an even lower posterior probability that the causal variants are shared between F2 and RBD (PPH4 = 0.0000002%), as well as between F2 and DLB (PPH4 = 2.27%), indicating one credible set per each trait pair. For the multiple causal variants assumption, colocalization between the four SNPs pairs of F2 and PD dropped to nearly zero for all four pairs. (Supplementary Table S3).

#### 2.2.3. eQTL–sQTL Colocalization Analysis

Given the complex nature of F2, which captures the shared genetic liability across RBD, PD, DLB, and MSA, we anticipated a broad pattern of enrichment involving multiple genes and brain regions. As expected, colocalization analysis via MetaXCan between F2 GWAS results and Brain eQTL and sQTL data revealed a diverse landscape of tissue-specific QTL effects. Significant brain-specific eQTLs were identified for 16 genes across 10 brain regions, while sQTL analysis, using a Bonferroni-corrected threshold, detected 27 variants in 10 brain regions. All detected significant signals are mapped to genes already identified in individual traits GWAS analyses. These results highlight the role of genomic variation onto transcriptomic alterations and the widespread involvement of both deeper brain structures, such as the basal ganglia and cerebellum, and outer cortical regions, including the frontal cortex, reflecting the complex genetic architecture underlying shared α-synucleinopathies genomic risk loci (Supplementary Table S2).

#### 2.2.4. ASSET

We retained SNPs when significant in ASSET two-sided test (Passet_combined < 1.6 × 10^−4^ = 0.05/306). Of the 306 significant SNPs identified in F2 mGWAS, 132 were still significant in the two-sided test (Supplementary Table S1). As we expected, none was predicted to be pleiotropic among all single traits, and this was plausible based on the genetic variability within F2 mGWAS.

**Figure 4:**
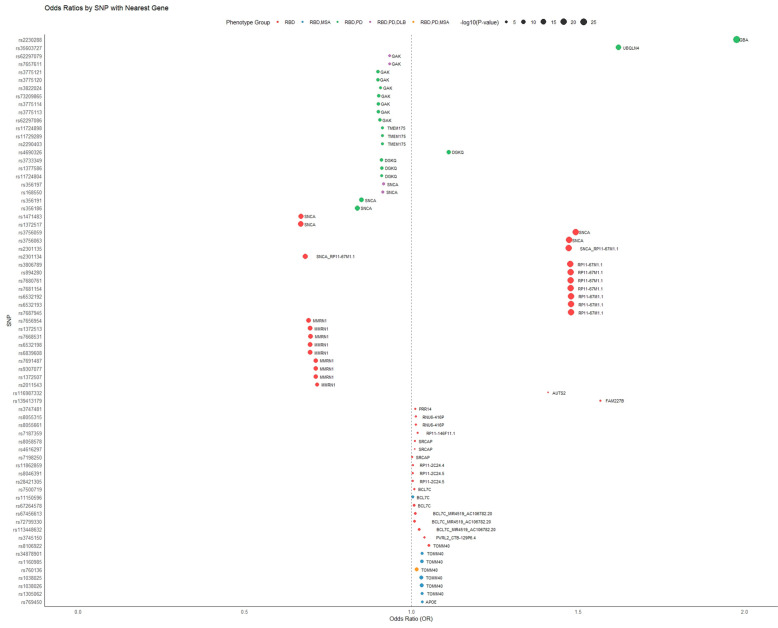
ASSET estimated pleiotropic SNPs associated with RBD traits. The Y axis displays SNPs identifiers, with top-down order from chromosome 1 to chromosome 22. The X axis displays Odds Ratio (ORs) value.

We sought to explore the pleiotropic contribution to F2, focusing specifically on SNPs predicted to be relevant in the RBD context. To this end, we prioritized SNPs either uniquely associated with RBD or exhibiting pleiotropic effects between RBD and one or more related traits. This approach allowed us to pinpoint potential genetic variants that may influence the trajectory of conversion from RBD to PD, DLB, or MSA. These SNPs were located in chromosomes 1, 4, 7, 15, 16, and 19. Predicted Odds Ratios (OR), along with their relative significance are depicted in Figure 4 (Supplementary Table S1).

We next decided to focus on the loci predicted to be associated exclusively with RBD to investigate whether RBD-predicted pleiotropic loci shared causal variants with other traits. To this end, we applied colocalization analysis to each trait pair. Loci on chromosomes 7, 15, and 19 were represented by only one or two SNPs each (rs116987332, rs139413179, rs3745150, and rs8106922, respectively), limiting their suitability for colocalization analysis. Thus, we selected one locus on chr4:90754292-90843509 and one on chr16:30666367-30898219, as these contained the highest number of predicted pleiotropic SNPs.

At the chromosome 4 locus, colocalization analysis suggested a high probability that a single causal variant was shared between F2 and RBD (PPH4 = 96.15%) and F2 and DLB (PPH4 = 96.6%), whereas the probability was much lower between F2 and PD (PPH4 = 4.43%). Under the multiple causal variants assumption, the posterior probabilities dropped to nearly zero for all analyzed traits.

In contrast, the chromosome 16 locus yielded different results. Under the single variant assumption, the probability of a shared causal variant was high between F2 and PD (PPH4 = 78%) and F2 and DLB (PPH4 = 96.34%), but much lower between F2 and RBD (PPH4 = 2.52%). Coloc.susie() failed to identify a credible set in both PD and RBD, so the analysis allowing multiple causal variants was performed only between F2 and DLB. The colocalization revealed a high PPH4 = 92.9% when considering LD between variants in the region (Supplementary Table S3).

#### 2.2.5. LDSC

**Figure 5:**
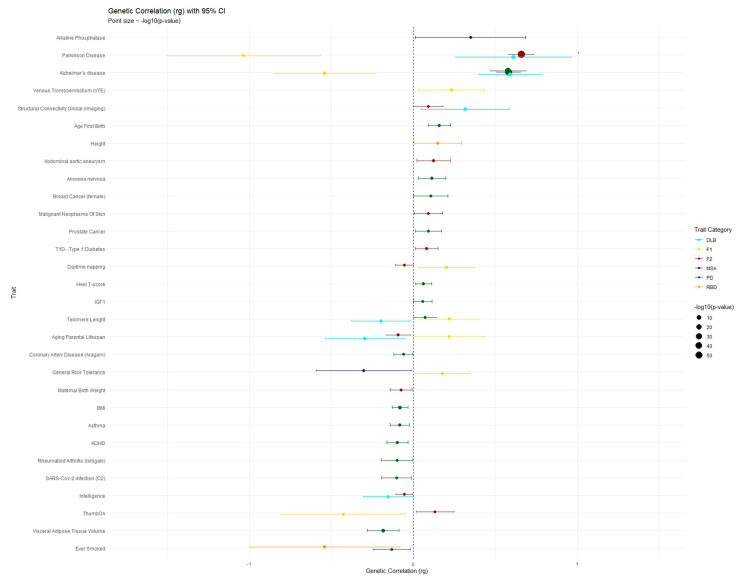
LDSC estimated correlation (rg). The Y axis represents single traits with significance correlation (*p* < 0.05) with one of each trait labeled in the legend. The X axis displays the level of correlation (rg).

To investigate the broader genetic architecture of F1, F2, and the original single traits, we examined their genomic correlations with a set of 106 highly heritable traits from UK Biobank (available at https://zenodo.org/records/10515792, accessed on 3 December 2024). We retained correlations reaching significance at *p* < 0.05. As expected, the strongest associations were observed with neurodegenerative traits, such as PD and Alzheimer’s disease (AD) (Supplementary Table S4). The following plot represents the levels of correlations and their respective *p*-values (Figure 5).

We conducted TwoSample MR [28,29] analysis on F1 and F2 LDSC significant results to inspect potential causal associations. No results showed causal associations between trait-pairs.

#### 2.2.6. Mendelian Randomization

We conducted MR analysis evaluating the potential causal associations between F1 or F2 with brain neuroanatomical correlates. Specifically, we tested the hypothesis that F1 or F2 instrumental variables (IVs) have a direct causal effect on volumetric brain structure measurements at the cortical and subcortical levels.

Using the aseg dataset on global brain area volumes, we observed a causal association between F1 liability and decreased Corpus Callosum (CC) mid-anterior volume. The results were consistent across Inverse variance weighted (IVW) (β = −0.009, *p* = 0.06), Weighted mode (β = −0.0149, *p*= 0.015), and Weighted median (β = −0.0146, *p* = 0.005). To assess potential bias, we performed MR-PRESSO. After re-running MR without the outlier, the IVW estimate lost significance (*p* = 0.16). In contrast, the MR-Egger estimate became slightly more negative and reached statistical significance (β = −0.019, *p* = 0.044), keeping the intercept non-significant, suggesting that the outlier was attenuating the true causal effect estimate in the original analysis, and that horizontal pleiotropy is unlikely to be a major source of bias, despite some sensitivity to outlier removal (Supplementary Table S4 and Supplementary Files SF1 and SF2).

F2 revealed an opposite direction of effect compared to F1. Increased genetic liability for F2 was associated with larger volumes in CC mid-anterior (IVW: β = 0.0084, *p* = 0.02), CC posterior (IVW: β = 0.0069, *p* = 0.04), and mid-posterior (IVW: β = 0.0067, *p* = 0.05) regions (Supplementary Table S4 and Supplementary Files SF3–SF6).

Delving into single-region volumes, we found a significant causal effect of F1 IVs on the volume of the right accumbens area. The IVW approach indicated a significant negative association (β = −0.0084, *p* = 0.0076), supported by the Weighted median estimator (β = −0.0099, *p* = 0.0294). While MR-Egger and mode-based estimators did not reach statistical significance, their effect sizes were consistent with a negative association (Supplementary Table S4 and Supplementary File SF7).

As expected, F2 MR results on single regions defined in the aseg dataset were less consistent, showing nominal associations primarily in the cerebellar cortex and cerebellar white matter (Supplementary Table S4 and Supplementary Files SF8–SF11). These findings exhibited considerable heterogeneity, with evidence of horizontal pleiotropy, which was anticipated given the nature of F2, as it encompasses a combination of distinct trait disorders, each affecting different subcortical structures.

At the cortical level, F1 MR analysis revealed a consistent association between F1 liability and increased left precuneus volume (DKT IVW: β = 0.009, *p* = 0.002), as well as a reduction in both lingual gyrus volumes (DKT_left IVW: β = −0.009, *p* = 0.02; DKT_right IVW: β = −0.006, *p* = 0.06) (Supplementary Table S4 and Supplementary Files SF12–SF14).

As with the aseg analysis, F2 MR results at the cortical level were more sparse and less consistent. However, we observed an indicative association between increased F2 liability and a reduced right lateral occipital volume. Results were significant in less robust methods (DKT Weighted median: β = −0.01, *p* = 0.05), but the effect size was consistent across all methods (Supplementary Table S4 and Supplementary File SF15).

## 3. Discussion

REM sleep behavior disorder is widely recognized as the strongest prodromal hallmark of α-synucleinopathies, with a high conversion rate to PD, DLB, or MSA over time. Notably, patients with RBD-associated neurodegeneration exhibit a more severe clinical trajectory compared to those without RBD, experiencing greater motor, cognitive, and autonomic dysfunction [1,2,3,4,5]. Despite these well-established epidemiological observations, the extent to which iRBD represents a distinct genetic entity or simply an early manifestation of neurodegeneration remains unclear.

The aim of this study was to disentangle the genetic architecture of iRBD from broader neurodegenerative liability using GenomicSEM in conjunction with LDSC and colocalization analyses. LDSC revealed an almost perfect genetic correlation between RBD [30] and DLB GWAS [31], a finding that aligns with their strong clinical overlap. However, this near-complete genetic similarity raises concerns about potential confounding in the original RBD GWAS, suggesting that unrecognized DLB cases within the analyzed cohort may have contributed to the genetic signal attributed to RBD. Colocalization analyses further reinforced this hypothesis, demonstrating a high probability of sharing causal variants at the shared *SNCA* locus between RBD and DLB, while overlap with PD was weaker. These findings highlight the necessity of distinguishing iRBD-specific genetic contributions from those driven by concurrent neurodegeneration.

In this study, we leveraged the largest available GWAS summary statistics of RBD [30], PD [32], DLB [31], and MSA [33] to dissect the complex genetic architecture underlying RBD and its progression toward neurodegeneration. Using a GWAS-by-subtraction framework implemented via GenomicSEM, we decomposed the genetic signal into two orthogonal latent variables: F1, which primarily captures the iRBD-specific genomic variation, and F2, representing the shared genomic covariance among iRBD, PD, DLB, and MSA.

GenomicSEM decomposition successfully isolated a “pure” iRBD signal from the genomic influences linked to neurodegeneration. Despite this separation, a low yet detectable level of colocalization between F1 and PD indicates that a neurodegenerative residual signal persists within the iRBD-specific component. This residual overlap may reflect latent neurodegenerative processes already active in iRBD, where additional genetic or environmental modifiers ultimately determine phenoconversion risk.

Our results show that F1 encompasses a risk locus in the *SNCA* region, which has been previously associated with RBD, PD, and DLB. F2 highlights several loci scattered throughout the genome, including variants in the 5′ region of *SNCA* and its antisense transcript *SNCA-AS1*. These findings align with earlier observations that PD-associated signals predominantly map to the 3′ region, whereas DLB-associated signals are more common at the 5′ end [34,35,36].

Fine-mapping of F1 identified an independent significant SNP (rs356224) at the 3′ end of *SNCA*, located within the lncRNA RP11-115D19.1. Both in silico and in vitro evidence suggest its role as a key antisense regulator of *SNCA*, likely exerting effects distinct from those of the 5′ end *SNCA* antisense transcript (*SNCA-AS1*), or reflecting spatio-temporal differences in regulation. Indeed, in vitro characterization highlighted a brain-specific regulation of RP11-115D19.1, which was further supported by the strong correlation between *SNCA* and RP11-115D19.1 expression in autopsied neurodegenerative brain cortices. The functional cellular studies suggested a possible role as a repressor of *SNCA* protein expression [20]. Variations in its sequence could alter transcription factor recruitment, dysregulating its endogenous repressor role, and enhancing the deposition and accumulation of *SNCA* neurotoxic aggregates. This brain-specific regulatory role suggests that RP11-115D19.1 may contribute to region-dependent transcriptional control of *SNCA*, potentially influencing disease vulnerability in distinct neuroanatomical contexts.

These findings reinforce the central role of *SNCA* in iRBD, suggesting that this locus acts as the primary genetic determinant of iRBD and its eventual conversion to a neurodegenerative disorder. The *SNCA* locus, long recognized as a major contributor to PD and DLB, appears to serve as the genetic backbone of iRBD, upon which additional modifying factors influence the specific trajectory of phenoconversion. Future studies are essential to account for potential confounding factors, including environmental variables, which should be carefully controlled. Addressing these factors is crucial to improving the reliability and translational applicability of the results.

The integration of genomic and transcriptomic data underscored the importance of *SNCA* fine regulation at both eQTL and sQTL levels, particularly in the cerebellum, where increased *SNCA* mRNA expression may contribute to neurotoxic accumulation [36,37,38]. In addition, we detected nominal signals on chromosome 3 mapping to eQTL in *GNAT1* and *SEMA3F*, as well as to sQTL in *SEMA3F-AS1*.

Semaphorins, and in particular *SEMA3F*, have been studied and associated with axonal guidance for the proper development of the limbic system [39]. Mice model studies have established the role of *SEMA3F* in the regulation and maintenance of proper neuronal circuit architecture. Impaired *SEMA3F* activity has been linked to disrupted amygdaloid circuitry, leading to improper neural connections within the amygdala and between the amygdala and other limbic structures, all relevant structures in REM sleep circuitry and REM motor control [40].

The observed reduction in *APOE* expression in F1, compared to an increased expression of *APOE* in F2, likely reflected the effect of our latent variable decomposition. The *APOE* signal arises from two SNPs previously described in AD contexts [41,42,43,44], linking them to accelerated cognitive decline. This result indicates that *APOE*-mediated toxic mechanisms are more relevant to neurodegenerative progression and cognitive decline, as captured by F2, rather than to an early-stage iRBD pathology, further reinforcing its pivotal role in cognitive decline even in α-synucleinopathies context [45,46].

ASSET analysis of pleiotropic loci revealed a complex pattern: only a few SNPs were predicted to be pleiotropic in at least three of the four traits, primarily those mapped to *SNCA* and its antisense transcripts. In contrast, many SNPs appeared to exert pleiotropic effects limited to specific trait subsets.

Specifically, the locus on chromosome 4 reinforced the pivotal role of *SNCA* and its nearby lncRNAs in modulating shared RBD-neurodegenerative liability without significantly influencing the phenoconversion trajectory.

The chromosome 16 locus was particularly noteworthy, as it overlapped with genes previously implicated in neurodegenerative GWAS studies [47]. Colocalization analysis further supported this association, pinpointing the presence of multiple causal variants shared between RBD and DLB. SNPs in this region mapped to *PRR14*, *SRCAP*, and *BCL7C*—genes linked to brain function, neurodegeneration, and cognitive decline. *PRR14* plays a role in nuclear lamina organization and chromatin tethering. Alterations in nuclear lamina structure have been linked to age-related cognitive decline and neuronal vulnerability [48,49].

*SRCAP* is involved in chromatin remodeling and regulates the CREB (cAMP response element-binding protein) pathway, which is critical for memory retention and hippocampal neurogenesis. In AD, disruptions in CREB-related pathways contribute to amyloid-beta accumulation and neurodegeneration. Additionally, rare *SRCAP* mutations have been identified in late-onset AD in Caribbean Hispanic families [50,51].

The association of *BCL7C* with neurodegeneration and cognitive decline is less well characterized, but it resides within the 16p11 locus, which has been associated with multi-traits genome-wide analysis (MTAG) studies of PD, DLB, and AD [47]. *BCL7C* is implicated in chromatin remodeling and stress-response pathways, both recognized as key mechanisms disrupted in neurodegenerative [52].

Taken together, the near-perfect genomic correlation between RBD and DLB, along with the high probability of colocalize causal variants in the chromosome 16 locus, suggests that this region may contribute to the accelerated cognitive decline observed in RBD converter patients. Further research should focus on functional experiments to confirm or discard the specific role of these genes and variants in neurodegenerative context.

Our LDSC correlation analysis provided valuable insights into the genomic architecture of the two latent variables and their association with a set of highly heritable traits. We found positive genomic correlations between F1 and daytime napping, as well as with telomere length and parental lifespan, while F2 showed an opposite trend. We then explored whether these relationships were driven by a shared genomic architecture or a direct causal association. MR analysis did not yield any significant results, suggesting a shared global genomic architecture or an indirect association, rather than a causal one.

Mendelian Randomization revealed an association between increased F1 liability and reduced mid-anterior CC volume, expanding upon previous findings that mid-posterior CC atrophy is a hallmark of RBD. In RBD patients with mild cognitive impairment (MCI) a significant reduction in mid-posterior CC volume has been documented compared to both RBD non-MCI patients and healthy controls [53,54]. MR on mid-posterior and posterior CC volumes did not yield significant associations with F1 liability, but the effect sizes suggested a trend toward negative associations. Conversely, increased F2 liability was associated with increased CC posterior volumes, potentially reflecting an altered functional connectivity and altered interhemispheric communication, or early compensatory response through neuroplasticity or neuroinflammation [53,54].

Subcortical analysis identified the right accumbens as the most significantly affected region in F1, with increased liability correlating with reduced accumbens volume, consistent with established literature describing both volume contraction and decreased accumbens connectivity in iRBD patients compared to controls [55,56]. For F2, increased liability was linked to enlarged cerebellar volumes. Cerebellar volume and cerebellar metabolism alteration have been constantly described in association with RBD [57,58,59,60,61]. Although both increased and decreased cerebellar volumes have been reported in RBD and neurodegenerative cohorts, an increased cerebellar cortex volume in early stages of neurodegeneration (e.g., MCI) may indicate maladaptive neuroplasticity or dysfunctional reorganization of networks [57,58,59,60,61].

At the cortical level, F1 IVs were associated with reduced lingual gyrus volumes, a finding previously described in iRBD patients. This could be part of a broad cortical thinning, or an impaired connectivity [62] with other brain regions, such as the nucleus basalis of Meynert [63,64], potentially affecting memory performance, visual memory, and semantic processing that may contribute to cognitive symptoms in RBD patients [64]. An interesting association was also observed between increased F1 liability and the larger precuneus area, contrasting with the volume reductions commonly reported in neurodegenerative contexts [65,66]. This suggests potential alterations of the global functional connectivity that warrant further investigation [67,68].

As in aseg, F2 MR results are more widespread and influenced by the model itself. F2 MR results indicated a putative reduction in the lateral-occipital cortex. The occipital cortex has been studied and implicated in the regulation of sleep patterns in mice models [69]. Further research is warranted to assess its neuroanatomical correlates with α-synucleinopathies.

Overall, these findings support the notion that iRBD-specific genetic liability drives early neuroanatomical changes long before clinical conversion, revealing patterns of brain connectivity that may influence the direction of disease progression. These neurogenetic alterations hold strong potential as clinically actionable biomarkers, supporting the integration of polygenic risk score stratification and neuroimaging-based markers into early diagnostic frameworks for at-risk patients [70]. The early recognition of RBD, combined with the ability to trace a probable phenoconversion trajectory, could facilitate the development of personalized risk prediction models to identify high-risk individuals before clinical symptoms emerge. This approach will aid in early patient stratification, targeted disease monitoring, timely neuroprotective interventions, and selection of individuals for optimizing the design of clinical trials and improving their efficiency. These findings provide a translational roadmap for integrating genetic and neuroimaging markers into precision medicine strategies for neurodegenerative disorders. Future studies should focus on identifying, validating and optimizing these markers for clinical implementation and tailored treatment strategies.

### Limitations

Despite the insights gained from the study, several limitations should be acknowledged.

First, we included all available α-synucleinopathies GWAS, despite substantial differences in sample sizes between them. Specifically, while PD had a large sample size, the other traits, particularly MSA, were relatively lower powered. This discrepancy is likely reflected primarily in the LDSC analysis, contributing to non-significant *p*-values, an inflated out-of-scale correlation between RBD and DLB, and a very low estimated heritability for MSA. The latter, with an h^2^ Z score below 2 as suggested by the authors, would have been excluded from the analysis. Nevertheless, its biological link to RBD prompted us to include it in order to capture the full spectrum of genetic variability across α-synucleinopathies.

We recognize that these limitations could have biased the downstream results, particularly the near-perfect correlation between RBD and DLB calls for caution when interpreting these findings.

Additionally, to ensure convergence in the genomic model, certain constraints were imposed. For example, RBD, and F1, F2 residual variance were set to zero, which is reflected as latent variables that are, in this scenario, 100% heritable, which in turn, makes the Neff unintuitively very small. These model constraints and factor loadings can affect results such as the stronger relationship between DLB and F2 compared to PD, likely due to higher factor loading of DLB onto F2.

The main limitation in the GCTA-COJO analysis was the choice of reference background. Despite the GCTA team suggesting the use of a reference panel bigger than 1000 Genomes, this was used for ease of availability.

In genomic-transcriptomic integration analysis, only 63% to 67% of available SNPs in the models were used. We attempted to leverage imputation within the MetaXCan package, but saw no significant increase on this percentage, which remained between 64% and 67%.

Finally, our analysis was conducted on the European population. This restricts the generalization of the findings, as genetic and molecular underpinnings may differ across diverse populations. Future studies are needed to validate these results in other ethnic groups to ensure broader applicability.

## 4. Materials and Methods

### 4.1. Data Accession and Pre-Processing

The data used for the analysis are available in “Data availability” section.

All summary statistics, excluding DLB and MSA, were already aligned against hg19 genome reference. DLB and MSA summary data were mapped against hg19 genome assembly via UCSC liftover (https://genome-store.ucsc.edu/, accessed on 1 January 2024). Missing dbSNP annotations were retrieved via dbSNP v150 (https://academic.oup.com/nar/article/29/1/308/1116004?login=true, accessed on 1 January 2024).

For all datasets, the Number of effective sample size (Neff) was calculated according to the formula available in the GenomicSEM package v.0.0.5 (https://github.com/GenomicSEM/GenomicSEM, accessed on 1 February 2024). For PD datasets, proxy cases and proxy controls were treated as cases and controls for Neff calculation.

### 4.2. LDSC

LDSC software v1.0.1 [11,71] was used to first, munge all input summary statistics in order to remove duplicated SNPs and align A1/A2 (effect/non-effect alleles) alleles to a common standard reference, and in so doing, adjust effect allele frequency and relative signed statistics in order to harmonize the input datasets.

After munging processing, harmonized summary statistics were used to calculate single trait heritability and genetic correlations estimates using HapMap3 variants only. Pre-calculated LD scores for LDSC were derived from the 1000 Genome project Phase3 European reference population (https://data.broadinstitute.org/alkesgroup/LDSCORE/eur_w_ld_chr.tar.bz2, accessed on 1 January 2024).

### 4.3. GenomicSEM

We used GenomicSEM R package v.0.0.5 [9] to implement GWAS by subtraction modeling [8]. Summary statistics were munged, and heritability, covariance and genetic correlation were calculated as in LDSC analysis. Once the data were prepped, a two-factor model was designed to superimpose the absence of correlation between latent factors, named F1 and F2 (Figure 2). To evaluate a more relaxed correlation between F1 and F2 and to see how model fits changed with correlation changes, three alternative models were tested, allowing for correlation between F1 and F2 at 0.1, 0.2, and 0.3. All models were evaluated considering Comparative Fit Index (CFI) and Standardized Root Mean Squared Residual (SRMR) values. A good fit for the model should be CFI > 0.9 and SRMR < 0.1. Since the four tested models were comparable and there were no big differences in fit indices, the analysis was performed using the zero-correlation model to further stress a strong isolation of signals. Both latent factors were regressed to perform a GWAS analysis, and the results were used for downstream investigation. The Neff for latent factors has been calculated according to the formula provided in the supplementary materials of Demange et al. 2021 [6] (https://github.com/PerlineDemange/non-cognitive/tree/master, accessed on 1 February 2024). According to authors’ recommendations, we removed SNPs with highly significant Q-statistics pvalues, since it indicates that they are likely to mediate their effects trough single traits rather than the common factor, which instead was the focus of the F2 mGWAS.

### 4.4. Genomic Loci Definition

Genomic loci were defined with FUMA (https://fuma.ctglab.nl/, accessed on 10 September 2024). The default settings were used, and the 1000 Genomes Project Phase3 of European ancestry was used as a reference panel. SNPs with *p* < 5 × 10^−8^ and independent from each other at r^2^ < 0.6 were defined as independent significant SNPs. Lead SNPs, a subset of the independent significant SNPs, were defined if they were independent from each other at r^2^  <  0.1. Genomic risk loci were identified by merging the LD blocks of independent significant SNPs that are closely located to each other (<250 Kb) [13]. The top lead SNP was defined as the SNP with the lowest *p*-value in a specific region. Functional annotations included: ANNOVAR v.1.6.2 categories (https://academic.oup.com/nar/article/38/16/e164/1749458, accessed on 10 September 2024), combined annotation-dependent depletion (CADD) scores (https://cadd.gs.washington.edu/), RegulomeDB scores (https://www.regulomedb.org/regulome-search/), eQTL from multiple sources: GTEx (https://gtexportal.org/home/), PsychENCODE (https://www.synapse.org/Synapse:syn4921369/wiki/235539), Common Mind Consortium-CMC (https://www.synapse.org/), eQTLgen (https://www.nature.com/articles/s41588-021-00913-z), and Chromatin interactions from multiple sources: HiC Giusti-Rodriguez et al. [72], PsychENCODE (https://www.synapse.org/Synapse:syn4921369/wiki/235539).

### 4.5. Fine-Mapping

We applied implemented fine mapping in FINEMAP [14] to retrieve credible sets for F1 and F2. An LD r matrix was extracted with Plink v1.0.7 [73] (https://zzz.bwh.harvard.edu/plink/, accessed on 1 February 2024) using the 1000 Genomes project Phase3 European reference panel. Analysis ran with default settings. We defined a set to be credible if the posterior probability (Ppr) was above 90%.

### 4.6. Conditional Analysis

GCTA-COJO v.1.94.2 (https://yanglab.westlake.edu.cn/software/gcta/#Overview, accessed on 1 May 2024) [15] was used to evaluate the significance of results after conditioning on the top hit and to evaluate a joint effect of significant SNPs in both the F1 and F2 GWAS results. Analysis ran with default settings. Again, 1000 Genomes Project Phase3 of European ancestry was used as the reference panel for estimating LD.

### 4.7. Colocalization

We focused our colocalization analysis exclusively at the *SNCA* locus (chr4:90393623-90893623), as it represents the primary genomic risk locus associated with RBD and the only locus shared between F1, F2, and each of the single traits, except MSA, which did not display any strong signals in such locus. First, an LD r score matrix was calculated using Plink v1.0.7 and 1000 Genomes European reference data. Then, using the coloc package [22], we performed both single causal variant assumption analysis (coloc.abf()) [23] and the improved approach, accounting for LD allowing multiple causal variant assumption, implemented in SusieR [24]. Default settings were used. Colocalization posterior probability (PP) threshold was set to 60%. PPs above this have been considered for inferences.

### 4.8. ASSET

Given that F2 GWAS is unable to account for pleiotropic effects of individual SNPs at the phenotypic level, we carried out an ASSET analysis [10] to explore the potential pleiotropy of SNPs in predicted optimal trait subsets. The analysis ran accounting for the correlation matrix between traits. We performed both a one-sided ASSET test assuming the same direction of association for all traits, and a two-sided ASSET analysis allowing for associations with opposite directions, to fully investigate the different association trajectory of SNPs on these traits. SNPs with P gwas < 5 × 10^−8^ were retained for ASSET analysis. We focused on two-sided test results only, where results were considered significant when P asset < 1.63 × 10^−4^ (0.05/306. 306 = Number of analyzed SNPs below P gwas).

### 4.9. eQTL–sQTL Colocalization Analysis

We leveraged the MetaXcan package (https://github.com/hakyimlab/MetaXcan, accessed on 1 May 2024) to integrate genomic and transcriptomic data [25] for expression Quantitative Trait Loci (eQTL) and splicing Quantitative Trait loci (sQTL). We used the S-PrediXcan function to predict gene expression from genotypes and used the predicted expression to test for associations with phen1otypes [26]. S-PrediXcan allows the usage of pre-compiled transcription models to compute association between omic features in a complex trait at single tissue level (https://github.com/stephenslab/mashr, accessed on 1 May 2024). We applied the MultiXCan function to compute omic associations, integrating measurements across tissues while factoring correlation [26]. In MultiXcan, we further focused on the GTEx v8 brain region only. We considered the results below the Bonferroni corrected threshold. eQTL genes tested were 22,535 and sQTL tests were 139,035. These set the Bonferroni significance to 2.2 × 10^−6^ and 3.6 × 10^−7^, respectively.

### 4.10. Mendelian Randomization

We used the TwoSampleMR R package [28,29] to perform MR analysis. For F1, we selected instrumental variables (IVs) by refining the package’s default settings. Since the top hit was slightly below Bonferroni significance, we relaxed the inclusion thresholds, selecting IVs with *p*-values <5 × 10^−6^ and performing clumping at r^2^ = 0.01 using the plink European reference panel.

For F2, which displayed more significant results, we applied a more stringent threshold for IVs selection. We used the default settings, selecting IVs with *p*-value <5 × 10^−8^ and clumped at r^2^ = 0.001.

After clumping to retain independent variables, we further assessed their F-statistics to ensure the strength of the selected IVs, applying an F-statistic threshold of 10. Any IV below this threshold were discarded. Additionally, we evaluated I^2^ statistics to determine the proportion of variance explained by the IVs rather than by noise. We considered I^2^ values greater than 0.75 as acceptable to minimize the inclusion of SNPs with high variance explained by noise.

Following these criteria, we retained 14 IVs for F1 (I^2^ = 0.76) and 91 IVs for F2 (I^2^ = 0.84).

The selected brain volumes outcomes were obtained from the IEU OpenGWAS project (https://gwas.mrcieu.ac.uk/, accessed on 1 September 2024), and specifically from the imaging segmentation dataset described in Elliot et al. 2018 [74]. We focused on two sets of brain imaging segmentation outcomes: the “aseg” segmentation dataset [75], which includes volumetric measurements of subcortical deep brain regions, and the “aparc” segmentation datasets which capture cortical brain structures. For aparc, we used both the Desikan [76] and the Desikan-Killiany-Tourville (DKT) atlases (https://trackvis.org/dtk/, accessed on 1 September 2024). We retained only results that were significant under both correction methods, ensuring robustness across different parcellation schemes. For simplicity, we referred to DKT-only results, since there were no big differences in the estimates.

Results were declared significant when they were consistent between the different MR methods. We used Simple mode, Weighted mode, Weighted median, Inverse Variance Weighted, and MR-Egger. For indicative results, we ran MR-Presso to detect potential outliers.

## 5. Conclusions

The GWAS by subtraction approach successfully disentangled iRBD-specific genetic liability from shared neurodegenerative risk, while simultaneously demonstrating that this risk is embedded within a broader neurodegenerative continuum. Our findings establish *SNCA* as the key genetic driver of iRBD, providing compelling evidence for the high likelihood of phenoconversion. Our findings highlight early structural brain alterations that may precede clinical onset, providing the foundation for genomic and imaging-driven risk stratification and prediction, and offering a framework for the development of neuroprotective strategies aimed at delaying or modifying disease progression.

## Data Availability

Summary statistics were mainly downloaded from GWAS catalog (https://www.ebi.ac.uk/gwas/, accessed on 1 January 2024): (a) RBD summary statistics study accession GCST9020420063; (b) DLB summary statistics study accession GCST9000139064; MSA summary statistics study accession GCST9040692465. PD summary statistics was taken from Nalls et al. 2019 [32], excluding Nalls et al. 2014, 23andMe post-Chang and colleagues and Web-Based Study of Parkinson’s Disease but including all analyzed SNPs, available at https://drive.google.com/drive/folders/10bGj6HfAXgl-JslpI9ZJIL_JIgZyktxn (accessed on 1 January 2024). Data generated during the study are available at https://doi.org/10.5281/zenodo.15041310.

## Supplementary Materials

The following supporting information can be downloaded at: https://www.mdpi.com/article/10.3390/ijms26083578/s1.
