# Supplementary material for: GWAS by Subtraction to Disentangle RBD Genetic Background from α-Synucleinopathies
Source: Int J Mol Sci. 2025 Apr 10;26(8):3578. doi: 10.3390/ijms26083578 (PMC12026788; doi:10.3390/ijms26083578)

Two sample MR report

Two sample MR report

F1 against aseg_global_volume_CC-Mid-Anterior || id:ubm-b-185

Date: **04 marzo, 2025**

Results from two sample MR:

method	nsnp	b	se	pval
MR Egger	13	-0.0195815	0.0086126	0.0440301
Weighted median	13	-0.0143464	0.0051891	0.0056977
Inverse variance weighted	13	-0.0064980	0.0046553	0.1627653
Simple mode	13	-0.0155080	0.0100000	0.1469107
Weighted mode	13	-0.0148121	0.0054558	0.0187841

Heterogeneity tests

method	Q	Q_df	Q_pval
MR Egger	18.77457	11	0.0652621
Inverse variance weighted	24.02001	12	0.0202139

Test for directional horizontal pleiotropy

egger_intercept	se	pval
0.016077	0.0091707	0.1073731

Test that the exposure is upstream of the outcome

snp_r2.exposure	snp_r2.outcome	correct_causal_direction	steiger_pval
0.0119755	0.0008733	TRUE	0.0009845

Note - R^2 values are approximate

Forest plot of single SNP MR

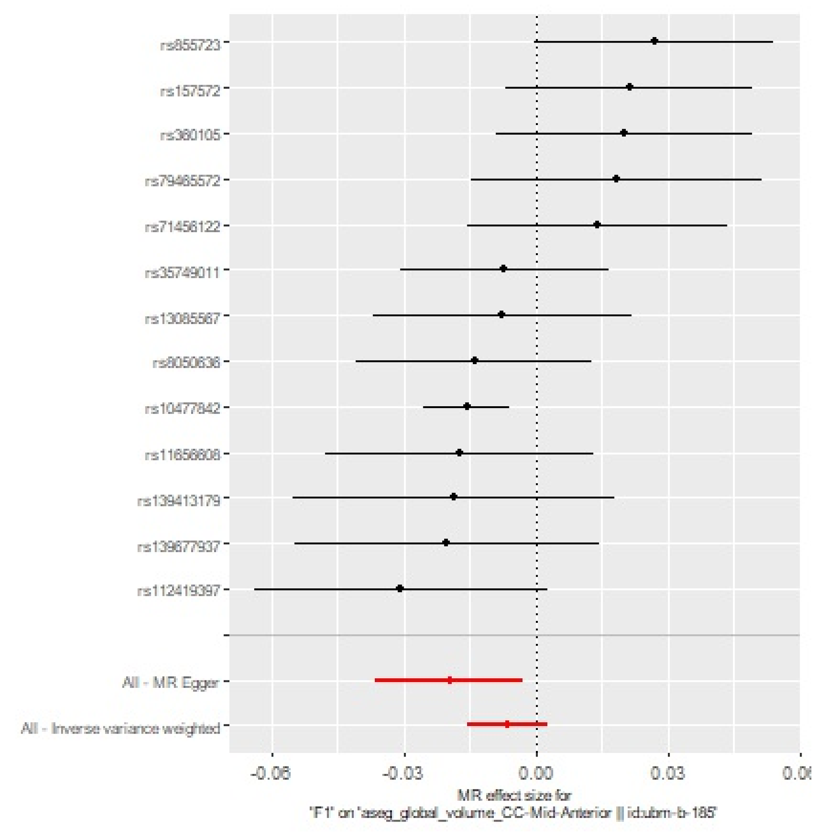

Comparison of results using different MR methods

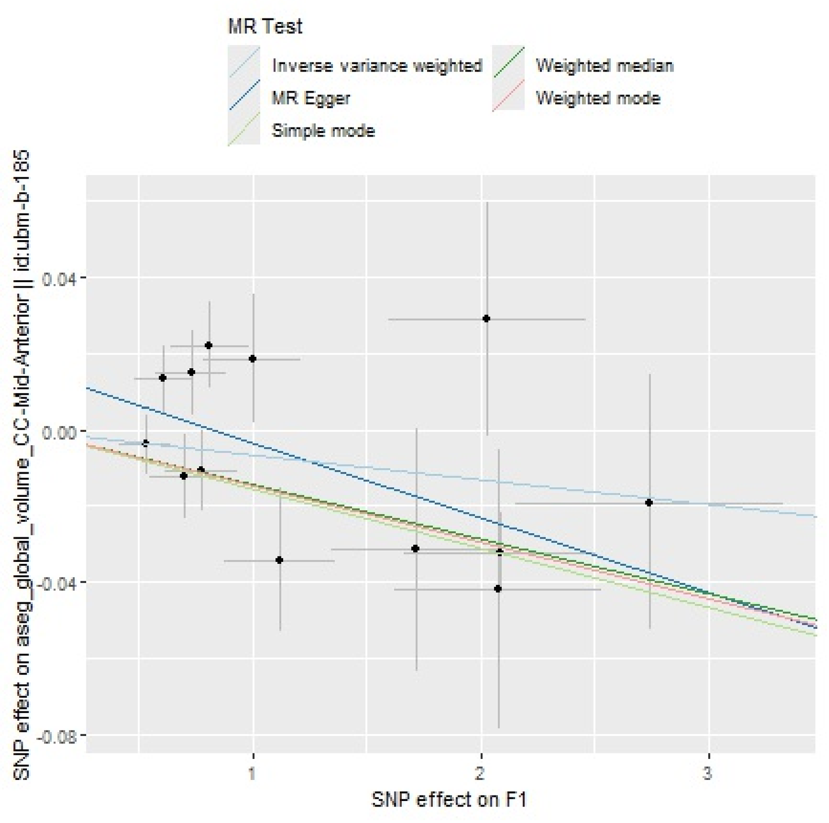

Funnel plot

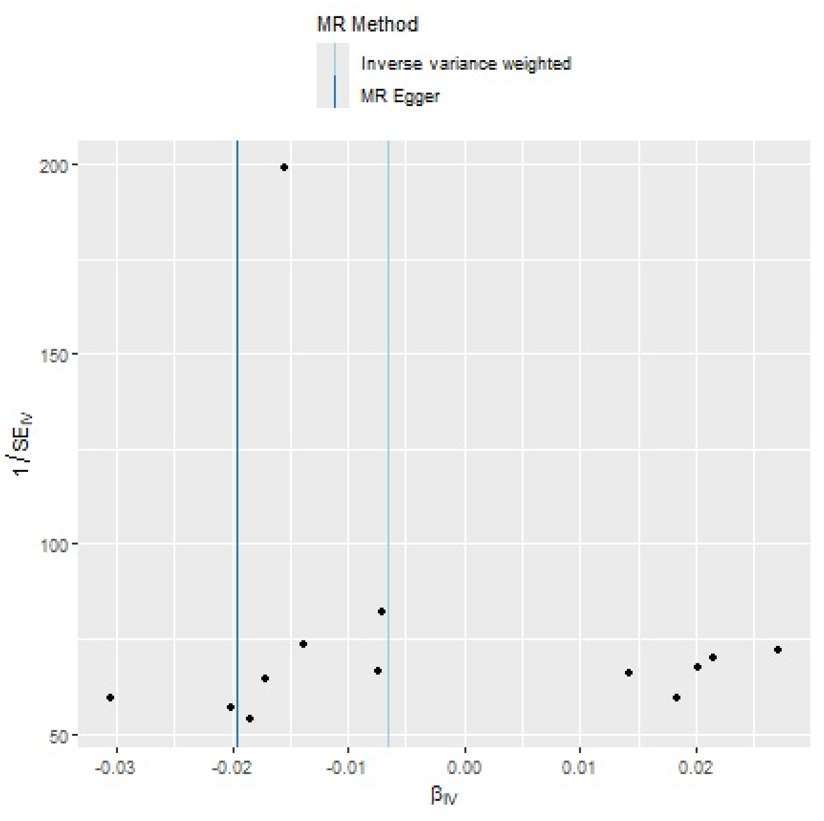

Leave-one-out sensitivity analysis

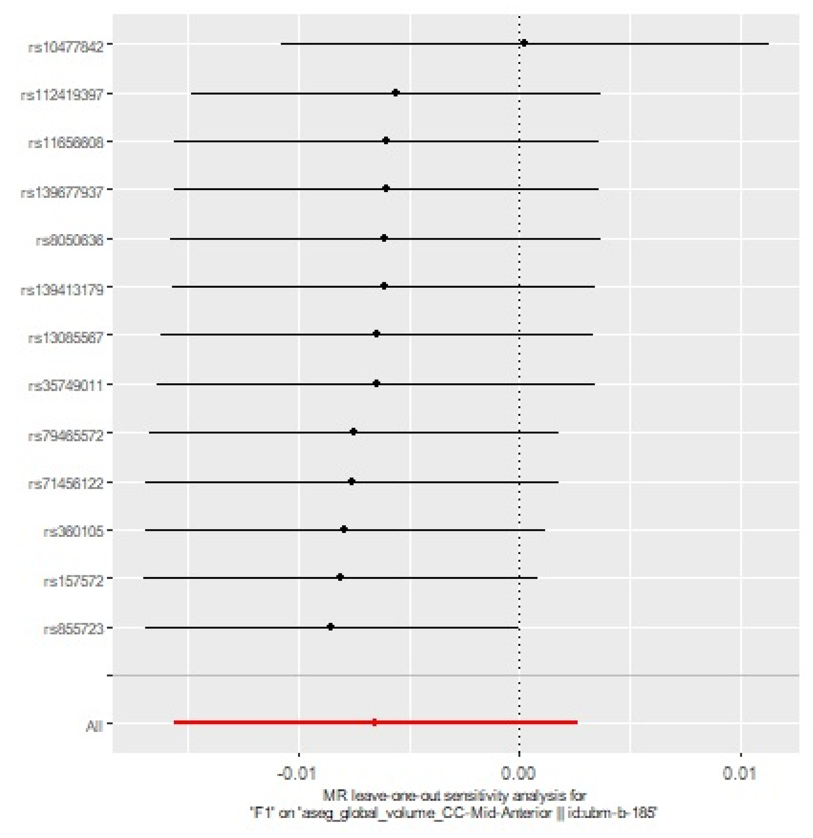

Supplement: Supplementary file 1 [file ijms-26-03578-s001.zip › ijms-3562618-supplementary/MR-PRESSO_TwoSampleMR.F1_against_asegglobalvolumeCCMidAnterior__idubmb185_SF2.pdf]
