# Supplementary material for: GWAS by Subtraction to Disentangle RBD Genetic Background from α-Synucleinopathies
Source: Int J Mol Sci. 2025 Apr 10;26(8):3578. doi: 10.3390/ijms26083578 (PMC12026788; doi:10.3390/ijms26083578)

Two sample MR report

Two sample MR report

F1 against aparc-DKTatlas_lh_volume_lingual || id:ubm-b-448

Date: **06 febbraio, 2025**

Results from two sample MR:

method	nsnp	b	se	pval
MR Egger	14	-0.0086020	0.0085212	0.3326685
Weighted median	14	-0.0123575	0.0047184	0.0088188
Inverse variance weighted	14	-0.0092212	0.0039482	0.0195133
Simple mode	14	-0.0107355	0.0080422	0.2048179
Weighted mode	14	-0.0123738	0.0052024	0.0334034

Heterogeneity tests

method	Q	Q_df	Q_pval
MR Egger	20.28410	12	0.0618991
Inverse variance weighted	20.29573	13	0.0880777

Test for directional horizontal pleiotropy

egger_intercept	se	pval
-0.0007271	0.0087654	0.9352578

Test that the exposure is upstream of the outcome

snp_r2.exposure	snp_r2.outcome	correct_causal_direction	steiger_pval
0.0123869	0.0009012	TRUE	0.0008652

Note - R^2 values are approximate

Forest plot of single SNP MR

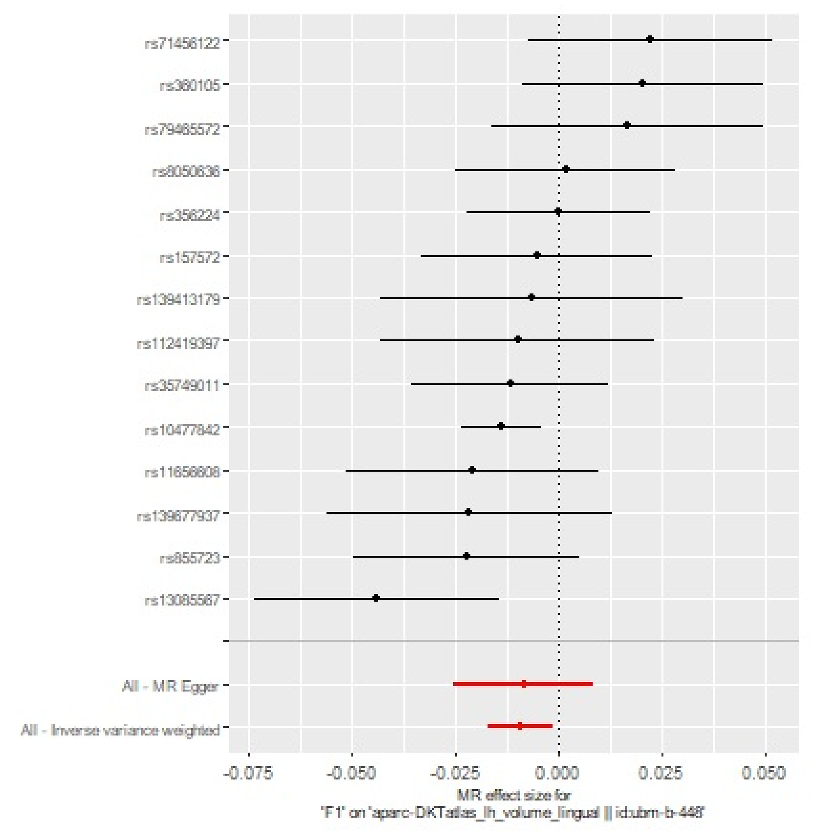

Comparison of results using different MR methods

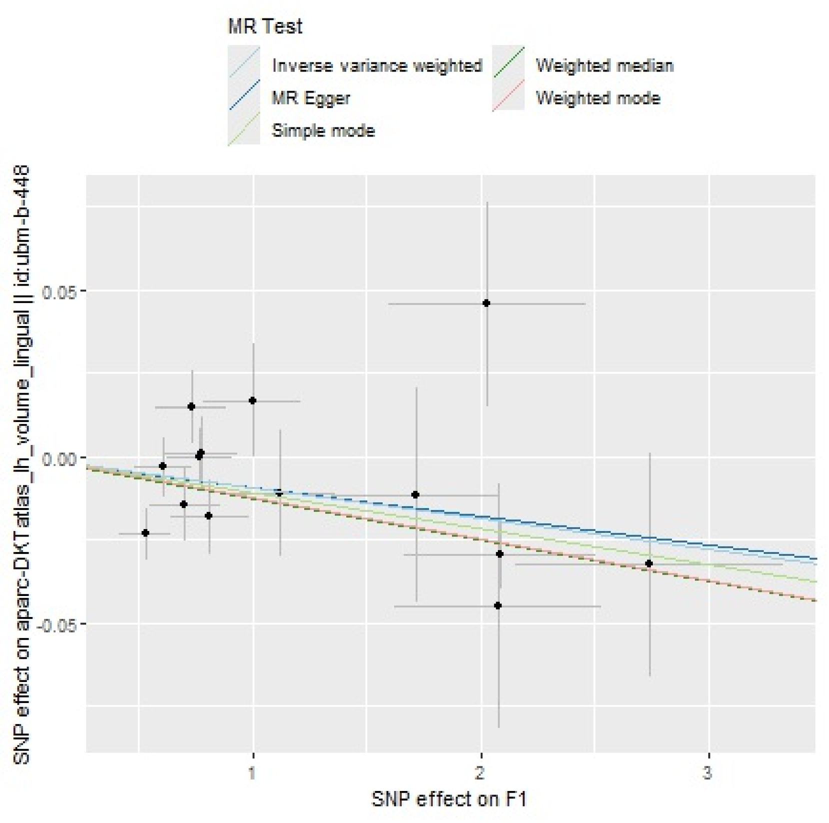

Funnel plot

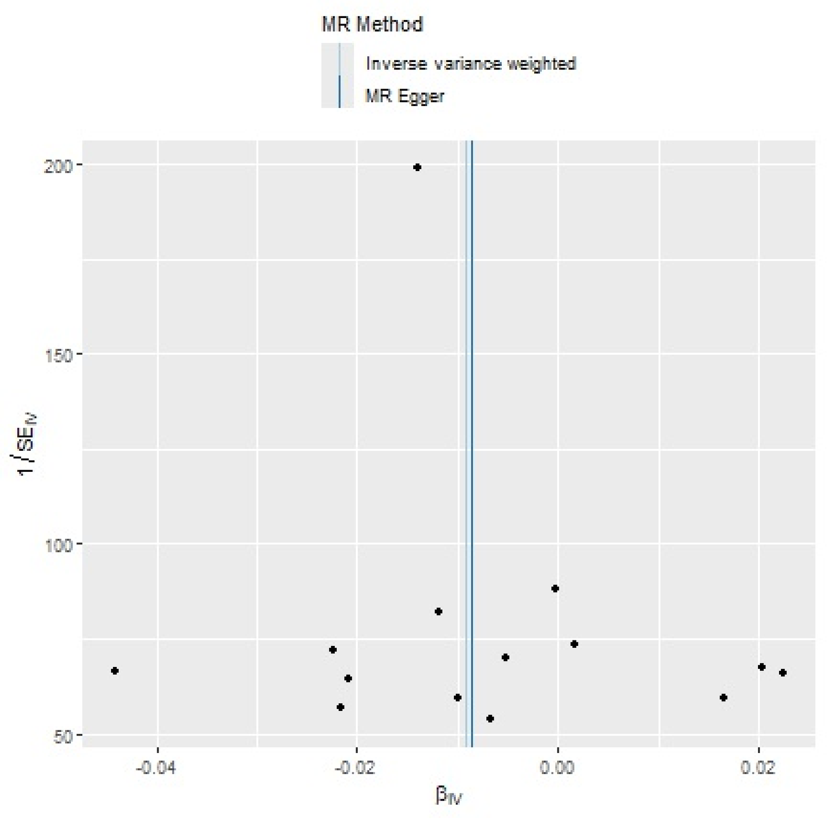

Leave-one-out sensitivity analysis

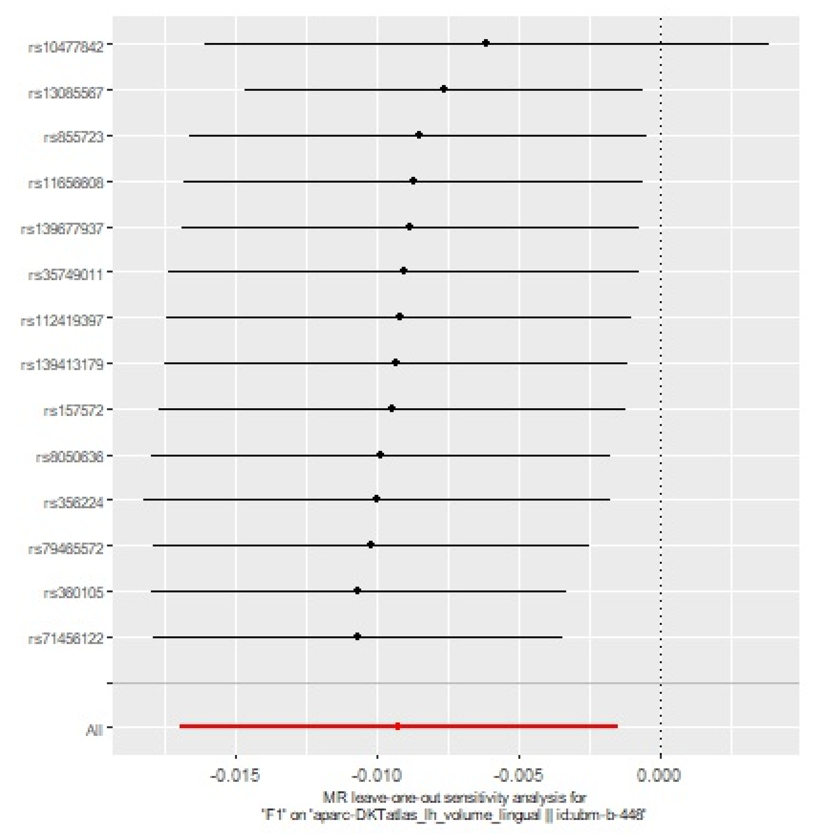

Supplement: Supplementary file 1 [file ijms-26-03578-s001.zip › ijms-3562618-supplementary/TwoSampleMR.F1_against_aparcDKTatlaslhvolumelingual__idubmb448_SF13.pdf]
