# Supplementary material for: GWAS by Subtraction to Disentangle RBD Genetic Background from α-Synucleinopathies
Source: Int J Mol Sci. 2025 Apr 10;26(8):3578. doi: 10.3390/ijms26083578 (PMC12026788; doi:10.3390/ijms26083578)

# Two sample MR report


# Two sample MR report


## F1 against aparc-DKTatlas_lh_volume_precuneus || id:ubm-b-460

Date: **06 febbraio, 2025**

### Results from two sample MR:

| method | nsnp | b | se | pval |
|---|---|---|---|---|
| MR Egger | 14 | 0.0108084 | 0.0068651 | 0.1413763 |
| Weighted median | 14 | 0.0090638 | 0.0047150 | 0.0545633 |
| Inverse variance weighted | 14 | 0.0096465 | 0.0031848 | 0.0024542 |
| Simple mode | 14 | 0.0127489 | 0.0075865 | 0.1167203 |
| Weighted mode | 14 | 0.0091595 | 0.0047728 | 0.0771928 |

### Heterogeneity tests

| method | Q | Q_df | Q_pval |
|---|---|---|---|
| MR Egger | 13.16429 | 12 | 0.3572136 |
| Inverse variance weighted | 13.20523 | 13 | 0.4320874 |

### Test for directional horizontal pleiotropy

| egger_intercept | se | pval |
|---|---|---|
| -0.0013642 | 0.0070617 | 0.8500483 |

### Test that the exposure is upstream of the outcome

| snp_r2.exposure | snp_r2.outcome | correct_causal_direction | steiger_pval |
|---|---|---|---|
| 0.0123869 | 0.0007046 | TRUE | 0.000515 |

Note - R^2 values are approximate

### Forest plot of single SNP MR

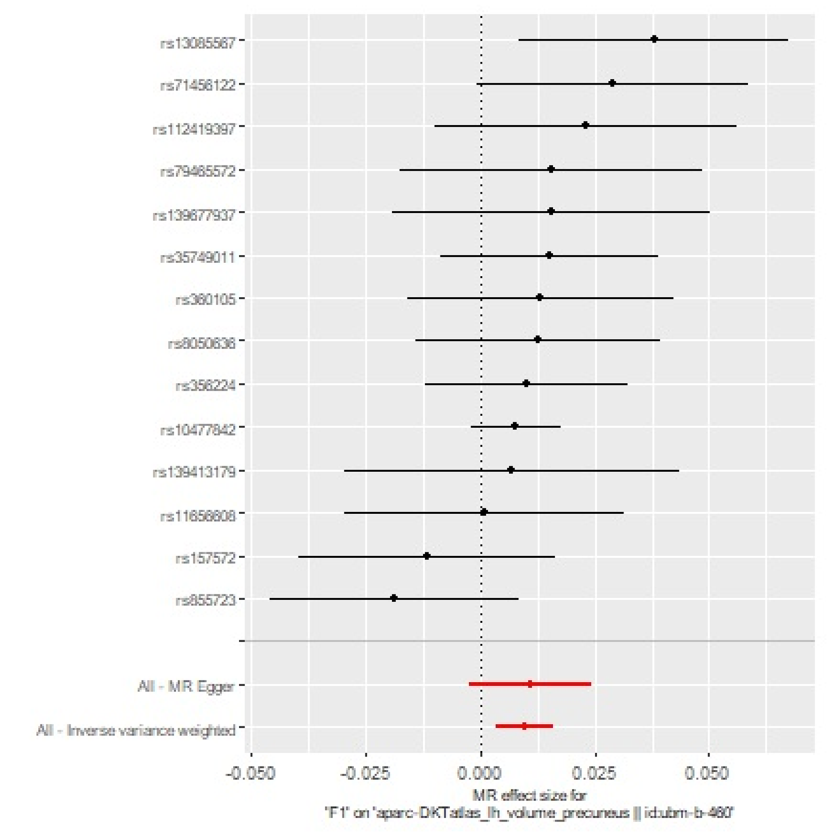


### Comparison of results using different MR methods

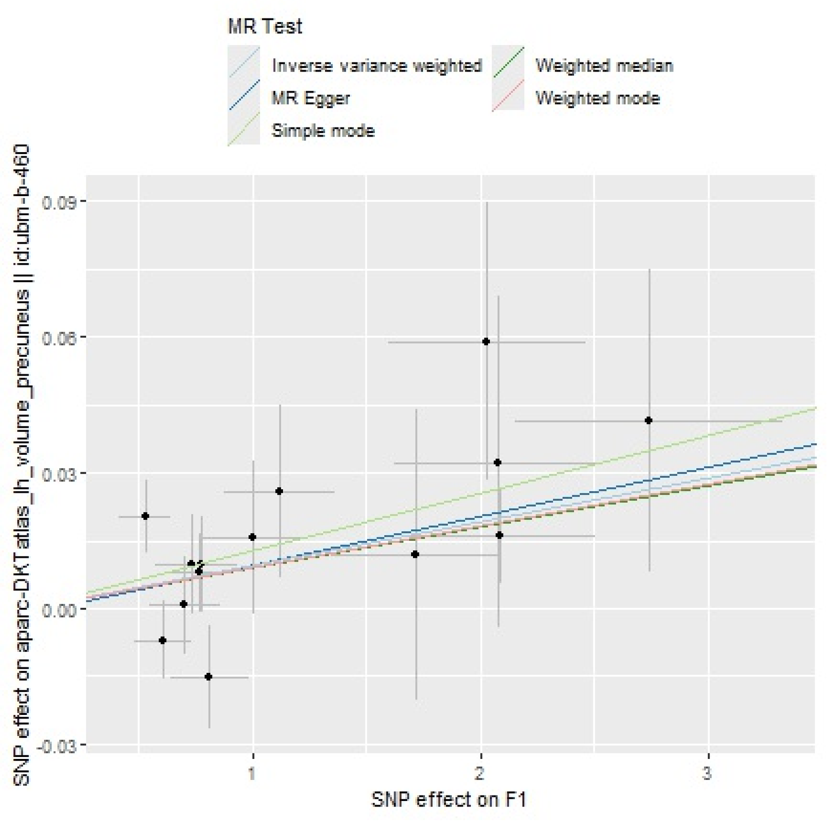


### Funnel plot

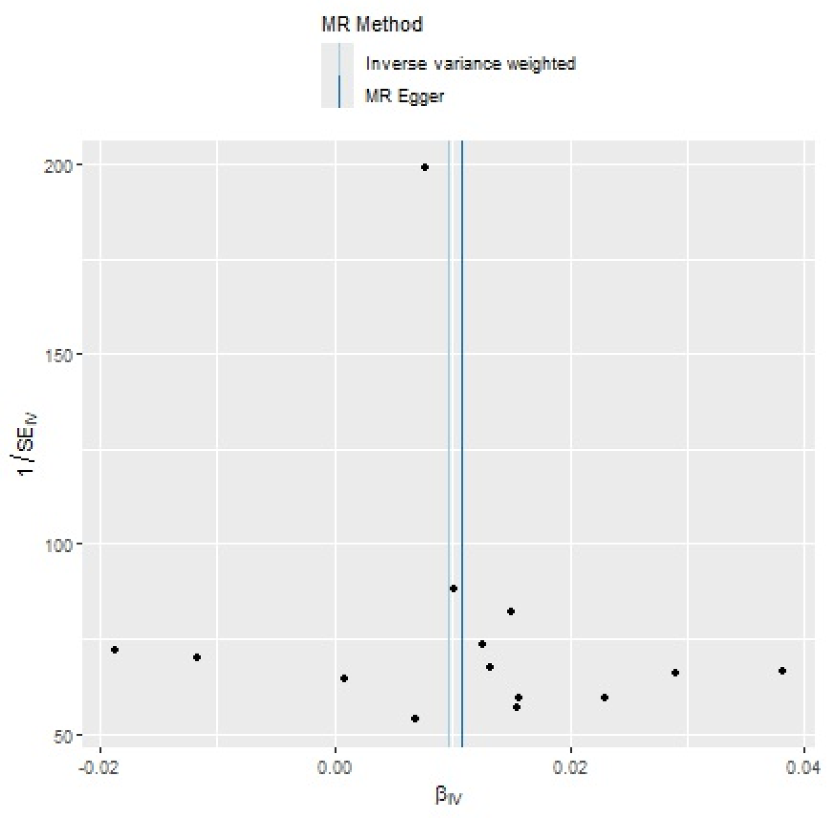


### Leave-one-out sensitivity analysis

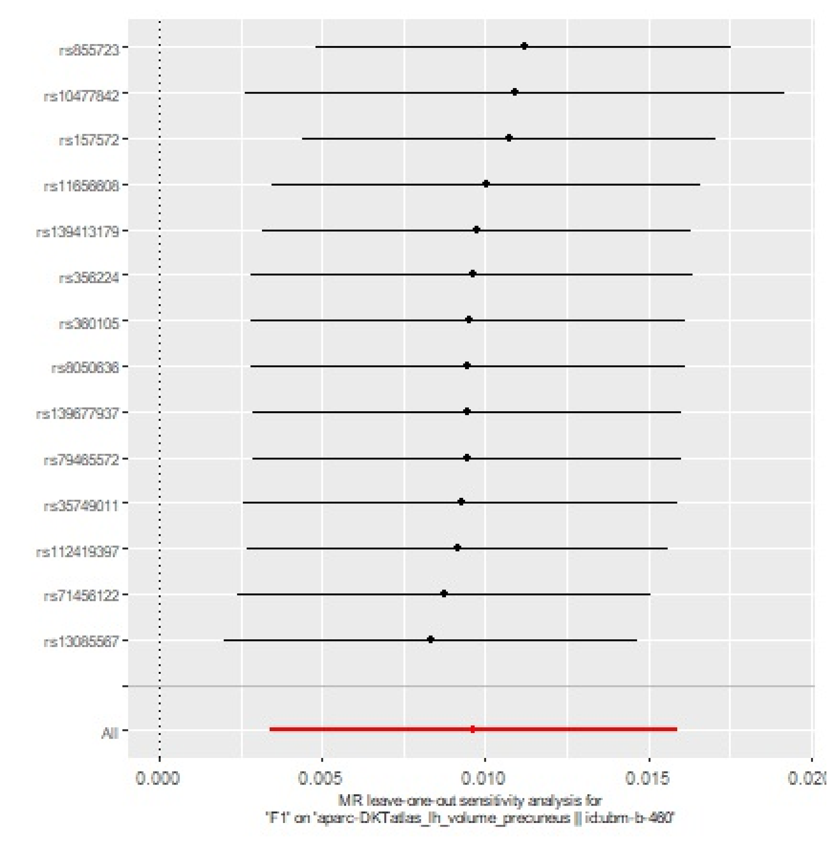

Supplement: Supplementary file 1 [file ijms-26-03578-s001.zip › ijms-3562618-supplementary/TwoSampleMR.F1_against_aparcDKTatlaslhvolumeprecuneus__idubmb460_SF12.pdf]
