# Supplementary material for: GWAS by Subtraction to Disentangle RBD Genetic Background from α-Synucleinopathies
Source: Int J Mol Sci. 2025 Apr 10;26(8):3578. doi: 10.3390/ijms26083578 (PMC12026788; doi:10.3390/ijms26083578)

Two sample MR report

Two sample MR report

F1 against aparc-DKTatlas_rh_volume_lingual || id:ubm-b-479

Date: **06 febbraio, 2025**

Results from two sample MR:

method	nsnp	b	se	pval
MR Egger	14	-0.0018570	0.0072719	0.8027682
Weighted median	14	-0.0071419	0.0045557	0.1169605
Inverse variance weighted	14	-0.0063300	0.0034368	0.0654995
Simple mode	14	-0.0199138	0.0094537	0.0551624
Weighted mode	14	-0.0079481	0.0049701	0.1337926

Heterogeneity tests

method	Q	Q_df	Q_pval
MR Egger	14.77055	12	0.2542248
Inverse variance weighted	15.37728	13	0.2843956

Test for directional horizontal pleiotropy

egger_intercept	se	pval
-0.0052517	0.0074802	0.49602

Test that the exposure is upstream of the outcome

snp_r2.exposure	snp_r2.outcome	correct_causal_direction	steiger_pval
0.0123869	0.0006065	TRUE	0.0003838

Note - R^2 values are approximate

Forest plot of single SNP MR

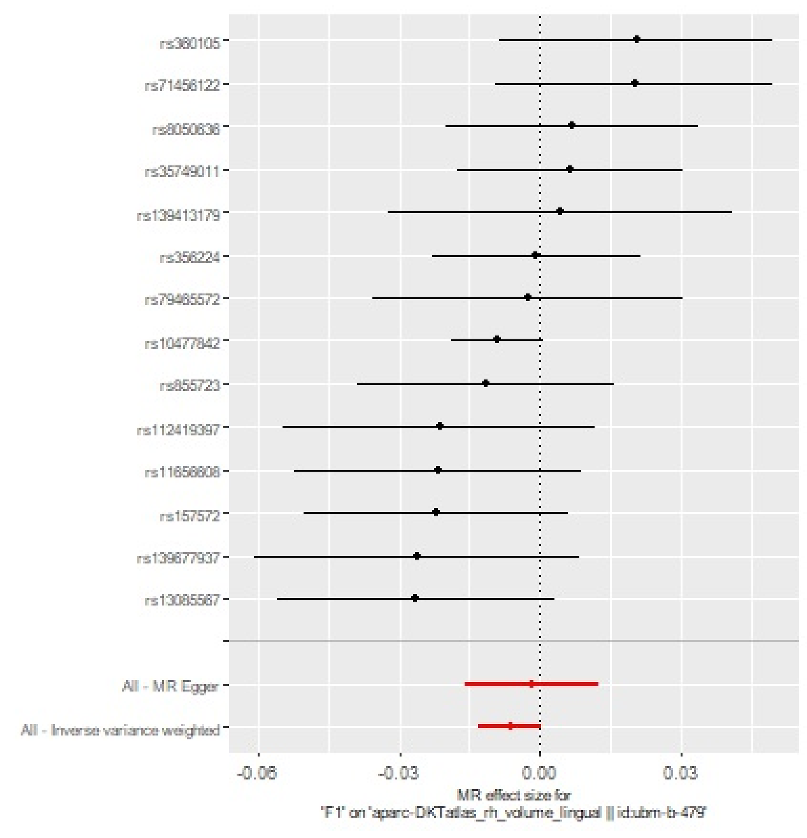

Comparison of results using different MR methods

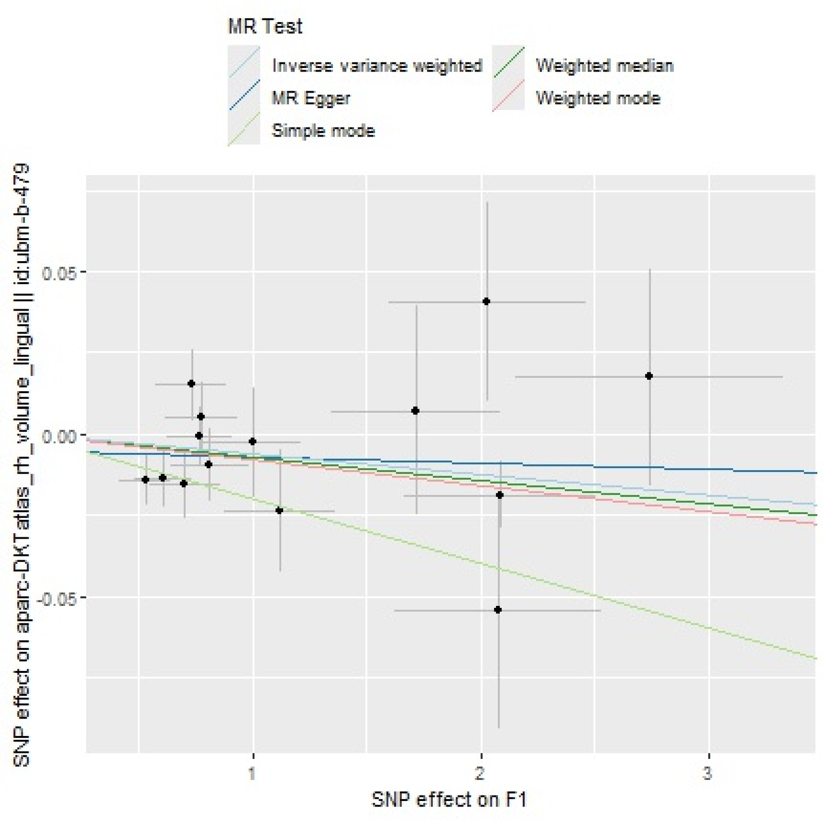

Funnel plot

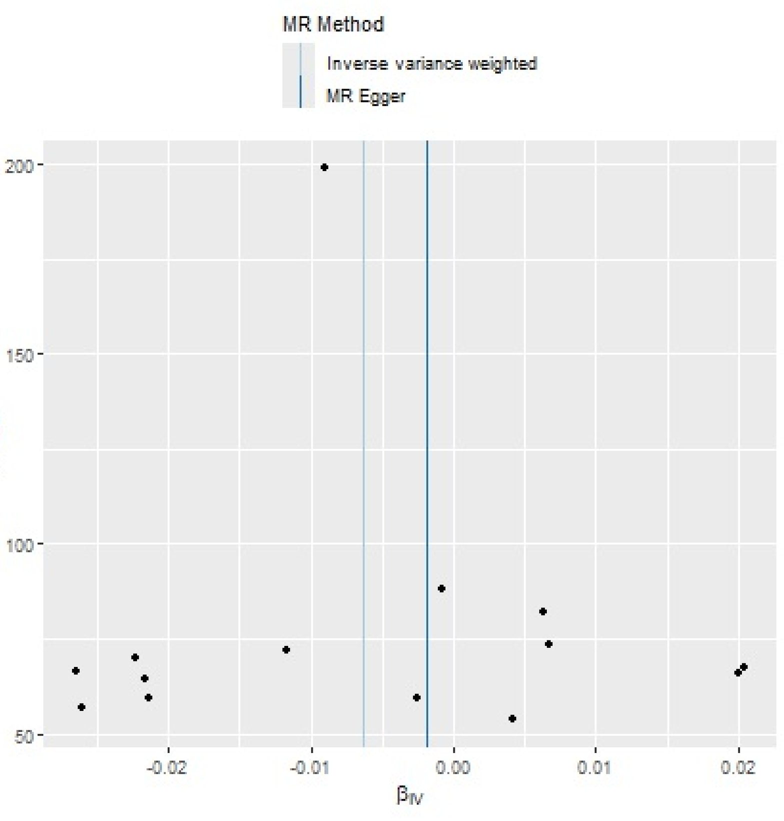

Leave-one-out sensitivity analysis

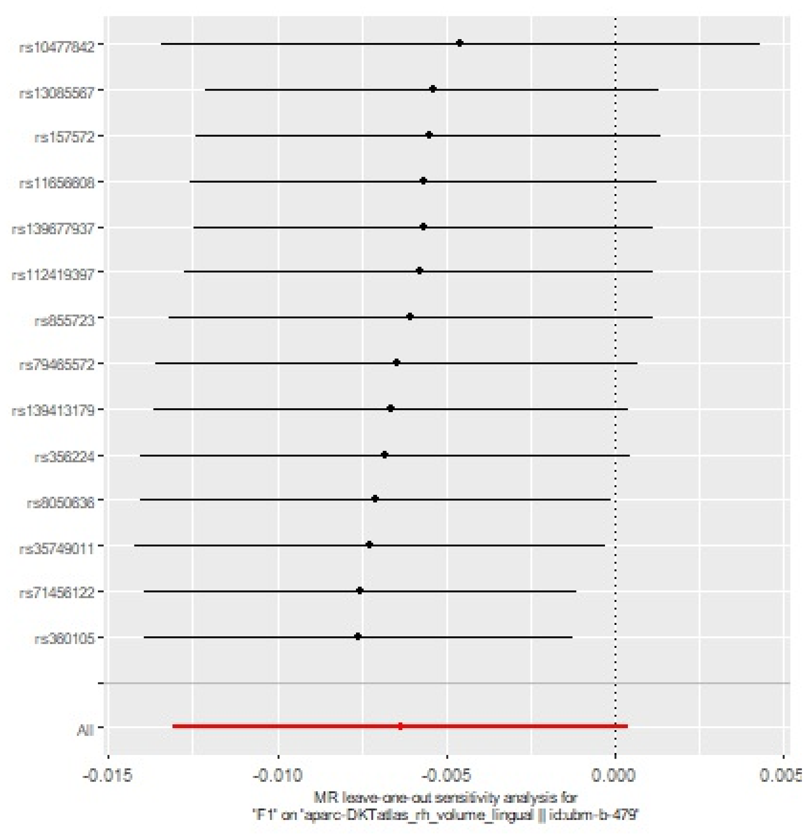

Supplement: Supplementary file 1 [file ijms-26-03578-s001.zip › ijms-3562618-supplementary/TwoSampleMR.F1_against_aparcDKTatlasrhvolumelingual__idubmb479_SF14.pdf]
