# Supplementary material for: GWAS by Subtraction to Disentangle RBD Genetic Background from α-Synucleinopathies
Source: Int J Mol Sci. 2025 Apr 10;26(8):3578. doi: 10.3390/ijms26083578 (PMC12026788; doi:10.3390/ijms26083578)

Two sample MR report

Two sample MR report

F1 against aseg_global_volume_CC-Mid-Anterior || id:ubm-b-185

Date: **07 febbraio, 2025**

Results from two sample MR:

method	nsnp	b	se	pval
MR Egger	14	-0.0171529	0.0104501	0.1266410
Weighted median	14	-0.0146132	0.0052519	0.0053950
Inverse variance weighted	14	-0.0091616	0.0049916	0.0664462
Simple mode	14	-0.0157484	0.0107830	0.1679014
Weighted mode	14	-0.0149495	0.0053619	0.0153758

Heterogeneity tests

method	Q	Q_df	Q_pval
MR Egger	30.50545	12	0.0023423
Inverse variance weighted	32.44215	13	0.0020649

Test for directional horizontal pleiotropy

egger_intercept	se	pval
0.0093826	0.0107495	0.3998807

Test that the exposure is upstream of the outcome

snp_r2.exposure	snp_r2.outcome	correct_causal_direction	steiger_pval
0.0123869	0.0012776	TRUE	0.0019517

Note - R^2 values are approximate

Forest plot of single SNP MR

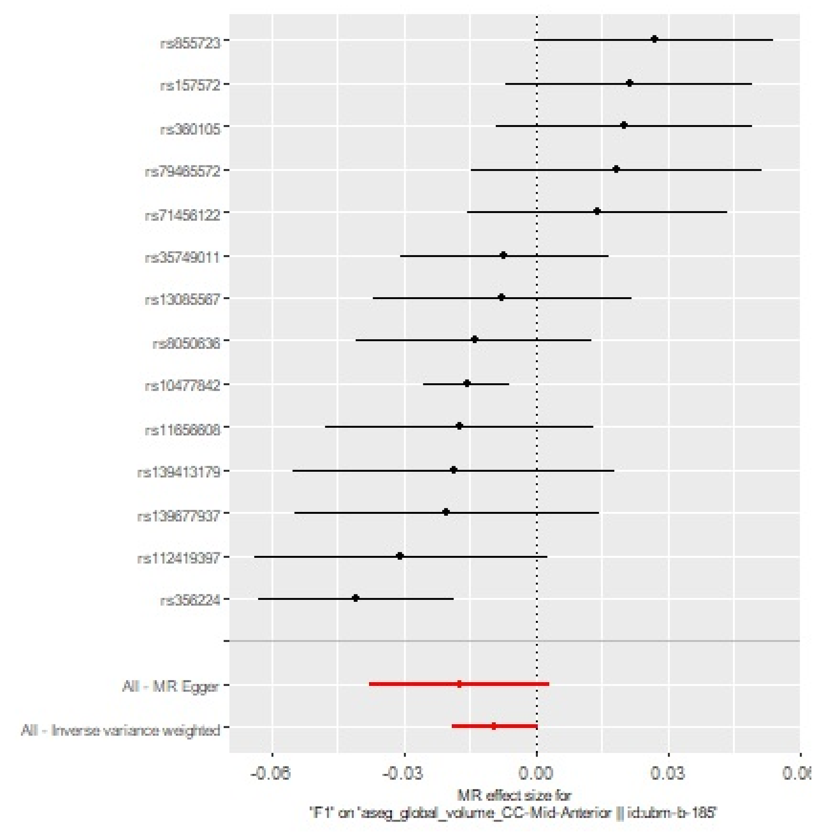

Comparison of results using different MR methods

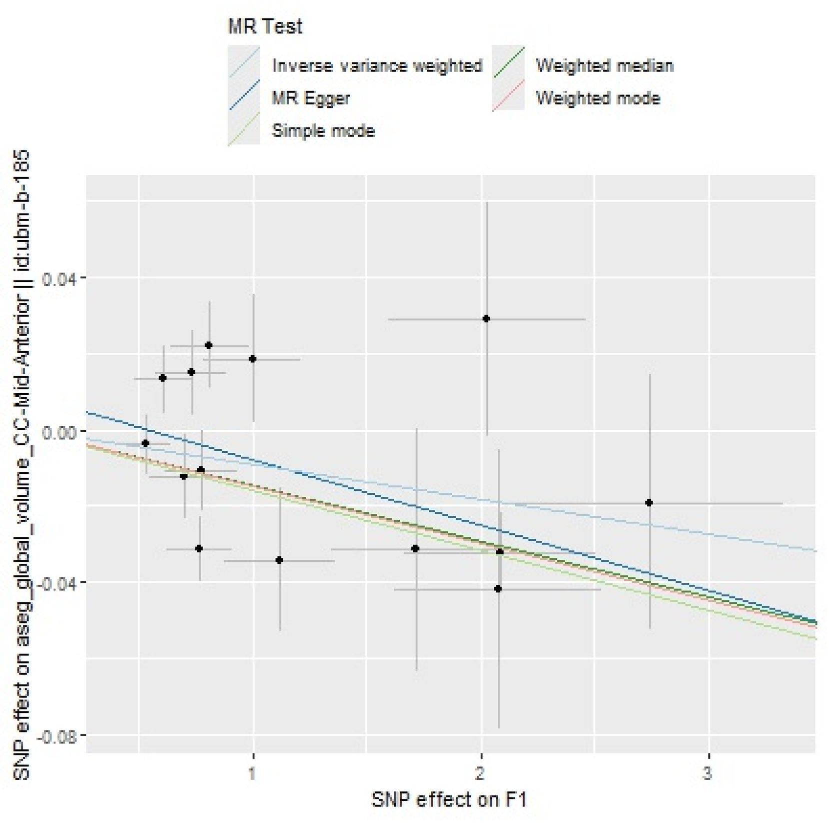

Funnel plot

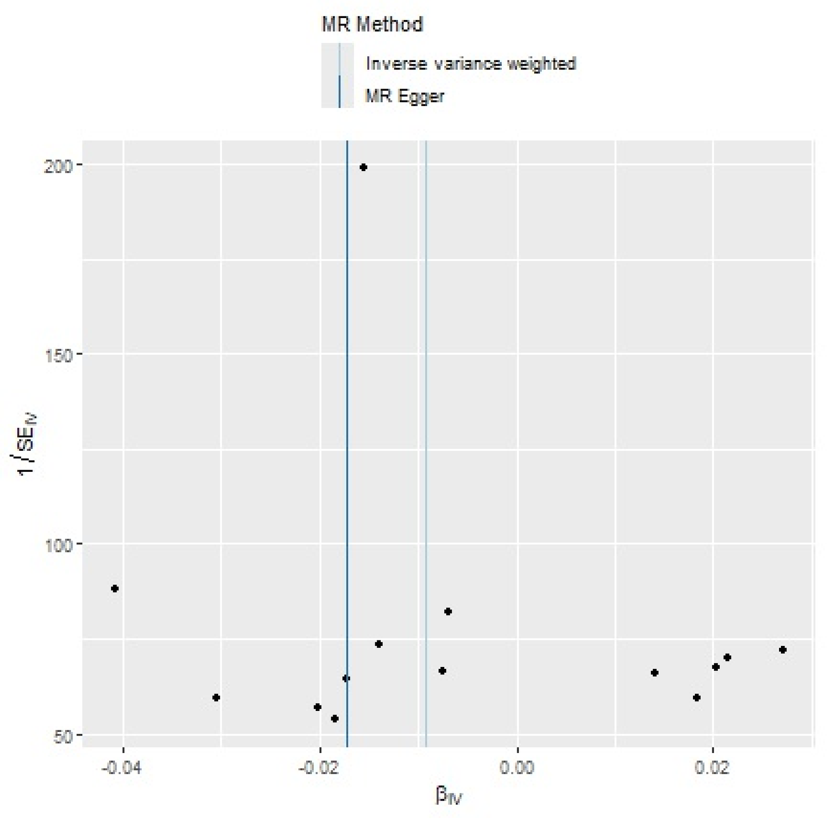

Leave-one-out sensitivity analysis

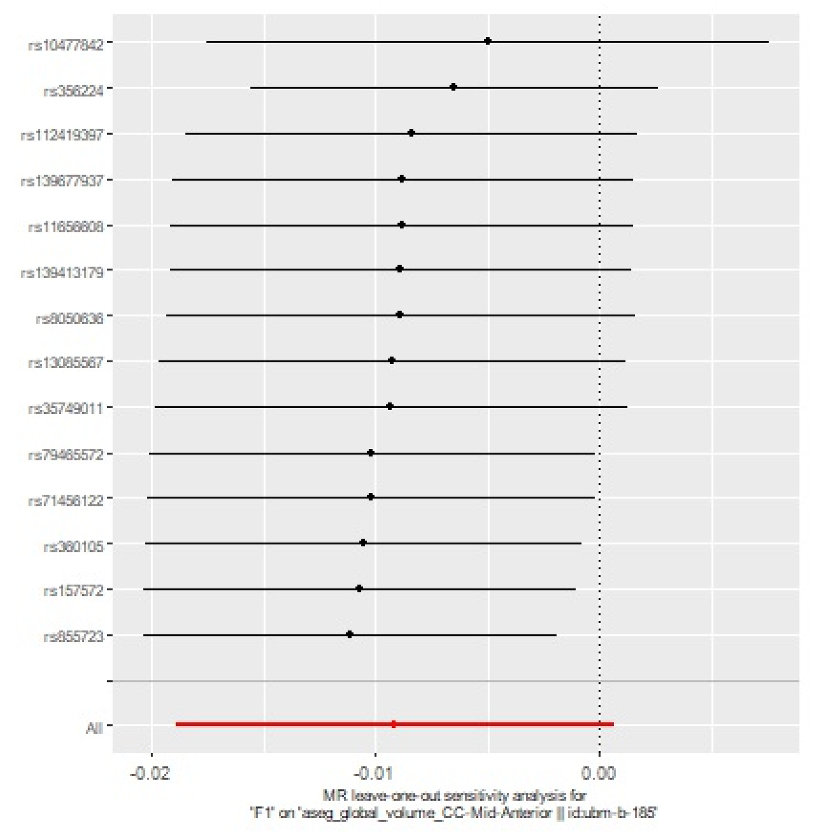

Supplement: Supplementary file 1 [file ijms-26-03578-s001.zip › ijms-3562618-supplementary/TwoSampleMR.F1_against_asegglobalvolumeCCMidAnterior__idubmb185_SF1.pdf]
