# Supplementary material for: GWAS by Subtraction to Disentangle RBD Genetic Background from α-Synucleinopathies
Source: Int J Mol Sci. 2025 Apr 10;26(8):3578. doi: 10.3390/ijms26083578 (PMC12026788; doi:10.3390/ijms26083578)

Two sample MR report

Two sample MR report

F1 against aseg_global_volume_CC-Mid-Posterior || id:ubm-b-183

Date: **04 marzo, 2025**

Results from two sample MR:

method	nsnp	b	se	pval
MR Egger	14	-0.0139120	0.0091379	0.1538064
Weighted median	14	-0.0092174	0.0050954	0.0704564
Inverse variance weighted	14	-0.0041510	0.0044872	0.3549211
Simple mode	14	-0.0086736	0.0109970	0.4444211
Weighted mode	14	-0.0109370	0.0054721	0.0669974

Heterogeneity tests

method	Q	Q_df	Q_pval
MR Egger	23.32623	12	0.0250806
Inverse variance weighted	26.21580	13	0.0159014

Test for directional horizontal pleiotropy

egger_intercept	se	pval
0.0114606	0.0093999	0.2461818

Test that the exposure is upstream of the outcome

snp_r2.exposure	snp_r2.outcome	correct_causal_direction	steiger_pval
0.0123869	0.000874	TRUE	0.0008091

Note - R^2 values are approximate

Forest plot of single SNP MR

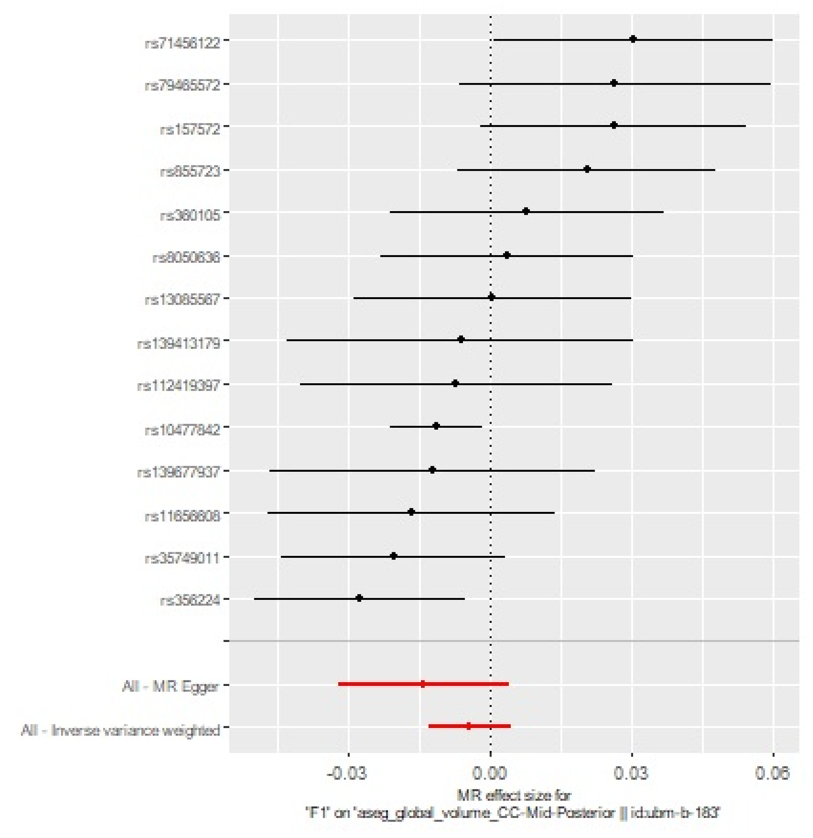

Comparison of results using different MR methods

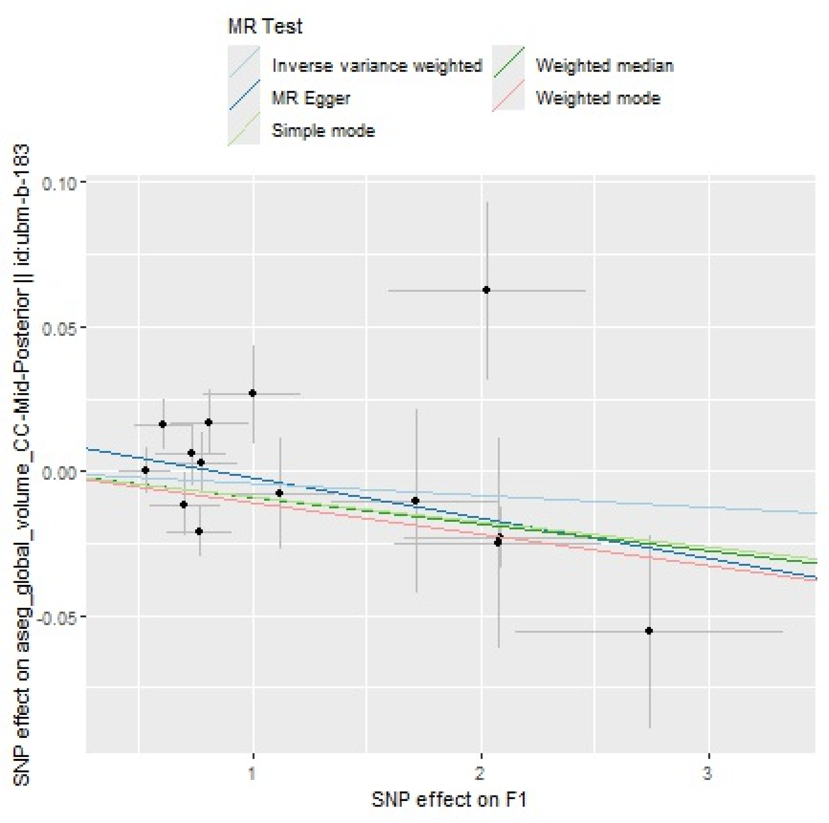

Funnel plot

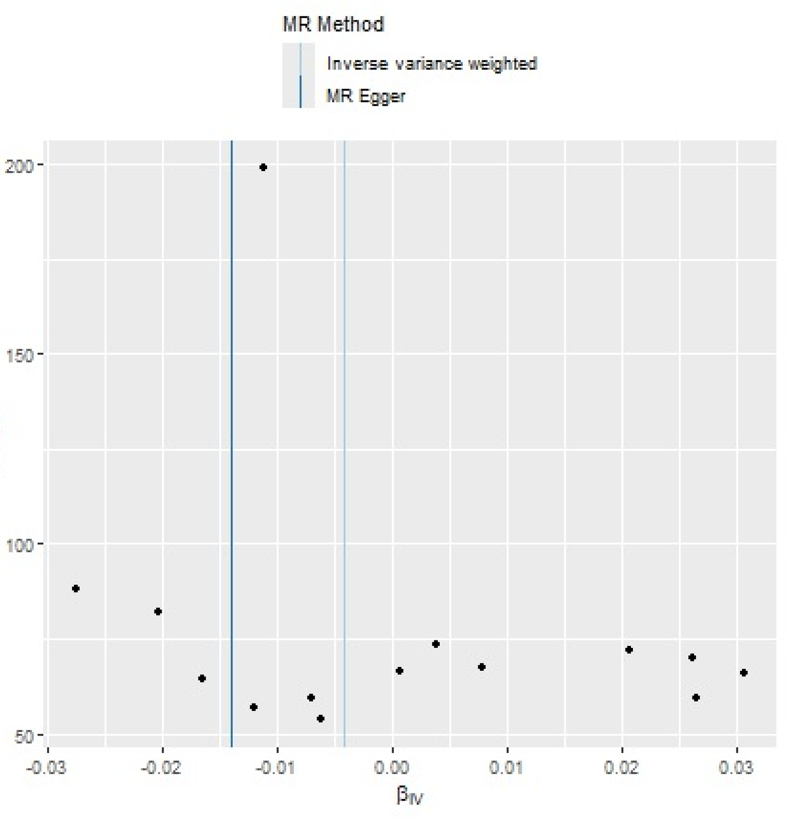

Leave-one-out sensitivity analysis

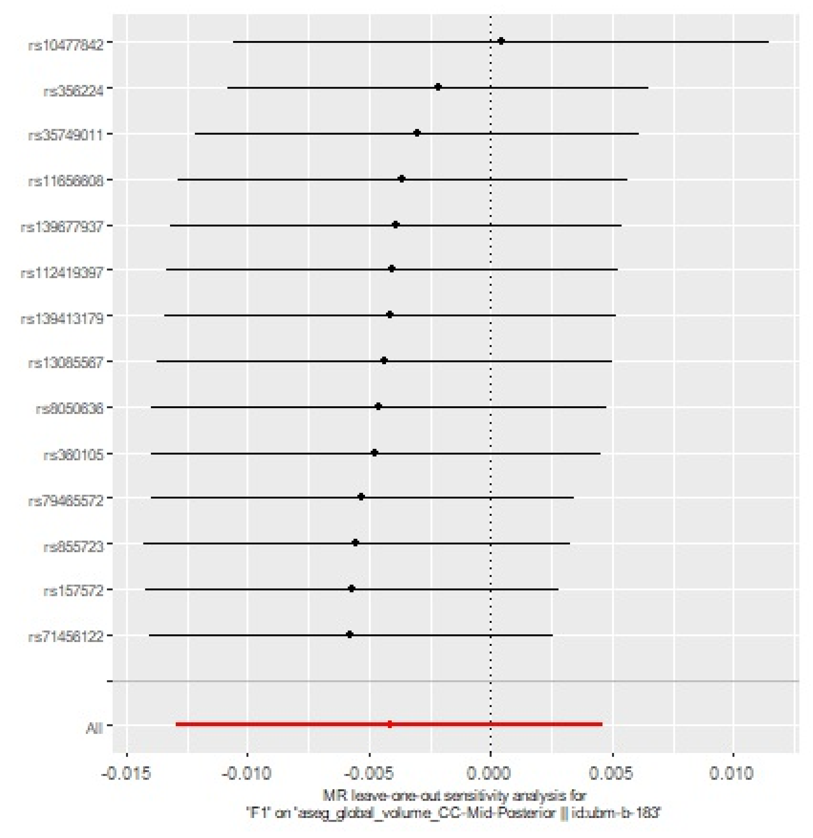

Supplement: Supplementary file 1 [file ijms-26-03578-s001.zip › ijms-3562618-supplementary/TwoSampleMR.F1_against_asegglobalvolumeCCMidPosterior__idubmb183_SF6.pdf]
