# Supplementary material for: GWAS by Subtraction to Disentangle RBD Genetic Background from α-Synucleinopathies
Source: Int J Mol Sci. 2025 Apr 10;26(8):3578. doi: 10.3390/ijms26083578 (PMC12026788; doi:10.3390/ijms26083578)

Two sample MR report

Two sample MR report

F1 against aseg_rh_volume_Accumbens-area || id:ubm-b-218

Date: **07 febbraio, 2025**

Results from two sample MR:

method	nsnp	b	se	pval
MR Egger	14	-0.0102082	0.0065546	0.1453393
Weighted median	14	-0.0098888	0.0045403	0.0294044
Inverse variance weighted	14	-0.0084355	0.0031600	0.0075968
Simple mode	14	-0.0063156	0.0078318	0.4345195
Weighted mode	14	-0.0097240	0.0051317	0.0805665

Heterogeneity tests

method	Q	Q_df	Q_pval
MR Egger	10.62579	12	0.5612311
Inverse variance weighted	10.72109	13	0.6341702

Test for directional horizontal pleiotropy

egger_intercept	se	pval
0.0020814	0.0067423	0.7628413

Test that the exposure is upstream of the outcome

snp_r2.exposure	snp_r2.outcome	correct_causal_direction	steiger_pval
0.0123869	0.0005583	TRUE	0.0003285

Note - R^2 values are approximate

Forest plot of single SNP MR

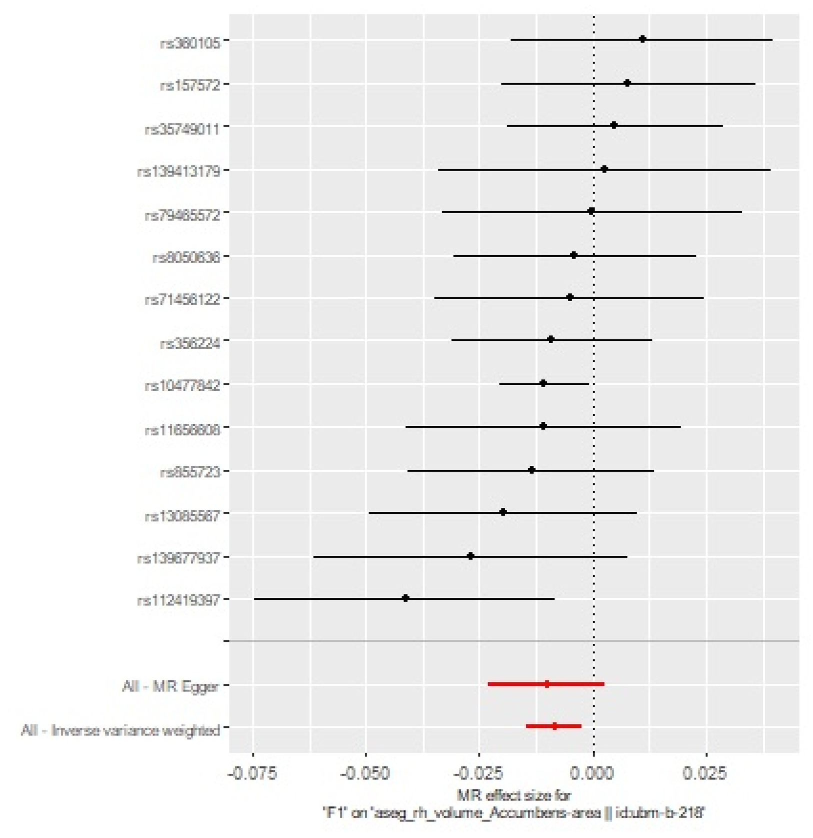

Comparison of results using different MR methods

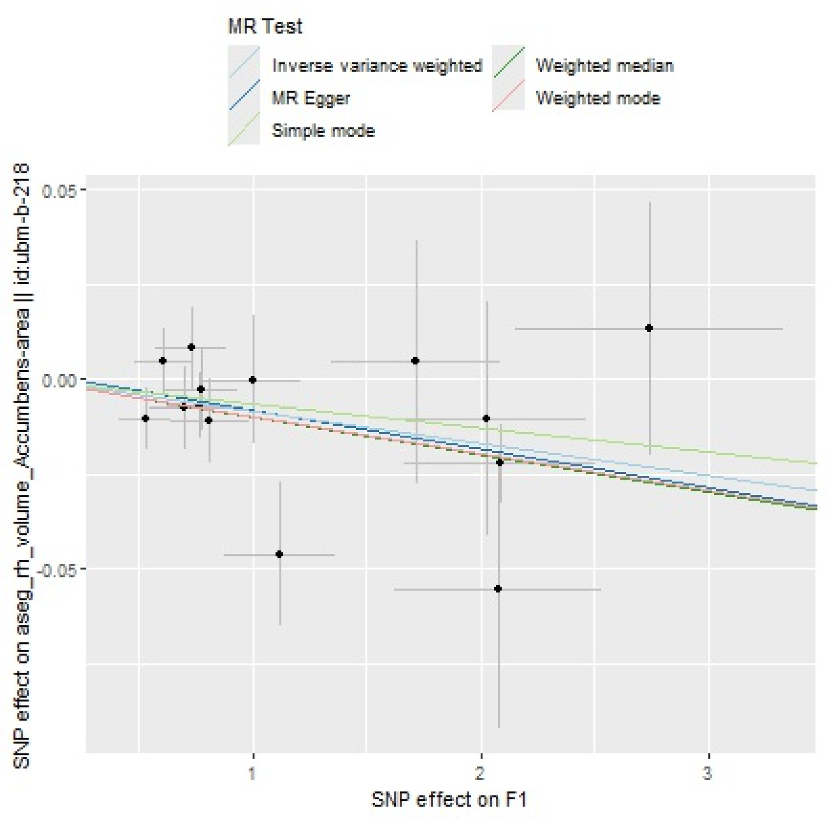

Funnel plot

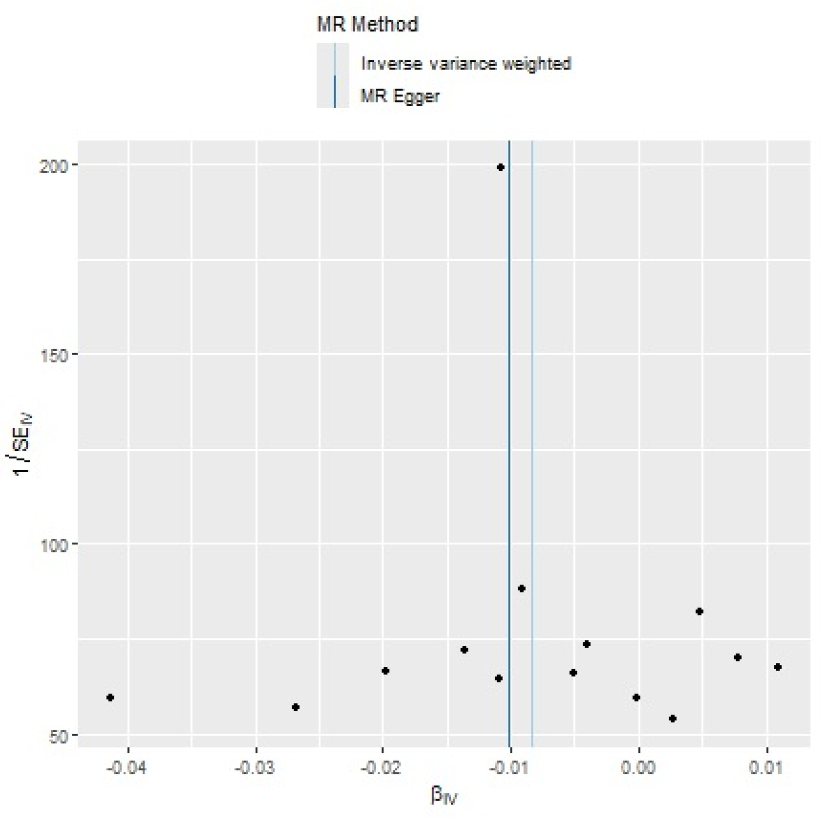

Leave-one-out sensitivity analysis

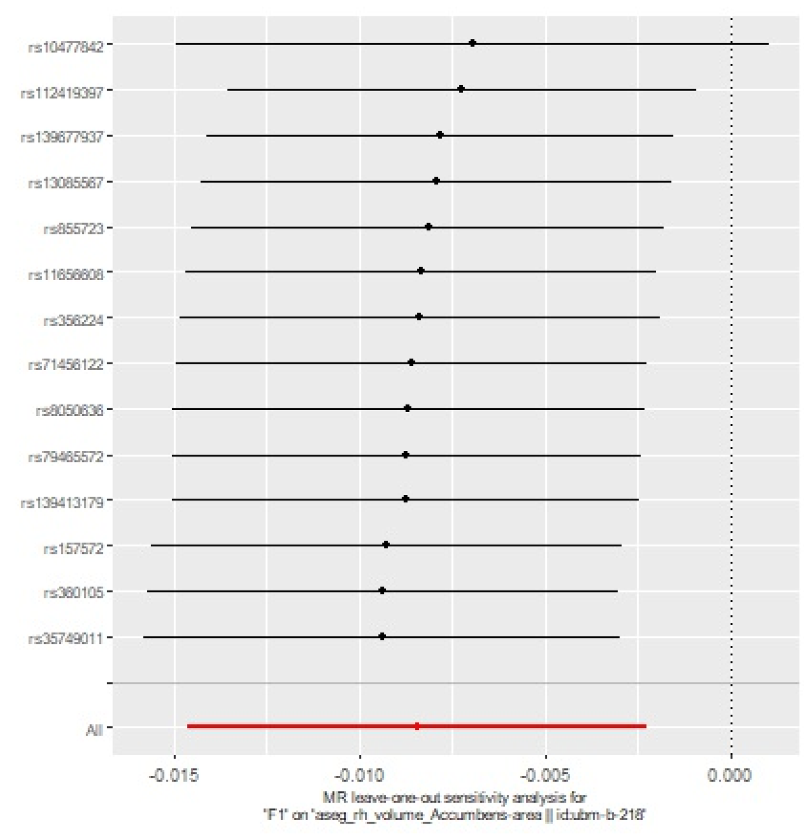

Supplement: Supplementary file 1 [file ijms-26-03578-s001.zip › ijms-3562618-supplementary/TwoSampleMR.F1_against_asegrhvolumeAccumbensarea__idubmb218_SF7.pdf]
