# Supplementary material for: GWAS by Subtraction to Disentangle RBD Genetic Background from α-Synucleinopathies
Source: Int J Mol Sci. 2025 Apr 10;26(8):3578. doi: 10.3390/ijms26083578 (PMC12026788; doi:10.3390/ijms26083578)

Two sample MR report

Two sample MR report

F2 against aparc-DKTatlas_rh_volume_lateraloccipital || id:ubm-b-477

Date: **10 febbraio, 2025**

Results from two sample MR:

method	nsnp	b	se	pval
MR Egger	91	-0.0015286	0.0071231	0.8305685
Weighted median	91	-0.0120994	0.0061100	0.0476745
Inverse variance weighted	91	-0.0054557	0.0034045	0.1090501
Simple mode	91	-0.0219525	0.0129002	0.0922618
Weighted mode	91	-0.0161458	0.0082061	0.0521985

Heterogeneity tests

method	Q	Q_df	Q_pval
MR Egger	90.67242	89	0.4307385
Inverse variance weighted	91.07457	90	0.4485028

Test for directional horizontal pleiotropy

egger_intercept	se	pval
-0.0026242	0.0041768	0.5314285

Test that the exposure is upstream of the outcome

snp_r2.exposure	snp_r2.outcome	correct_causal_direction	steiger_pval
0.00605	0.0029302	TRUE	0.0354075

Note - R^2 values are approximate

Forest plot of single SNP MR

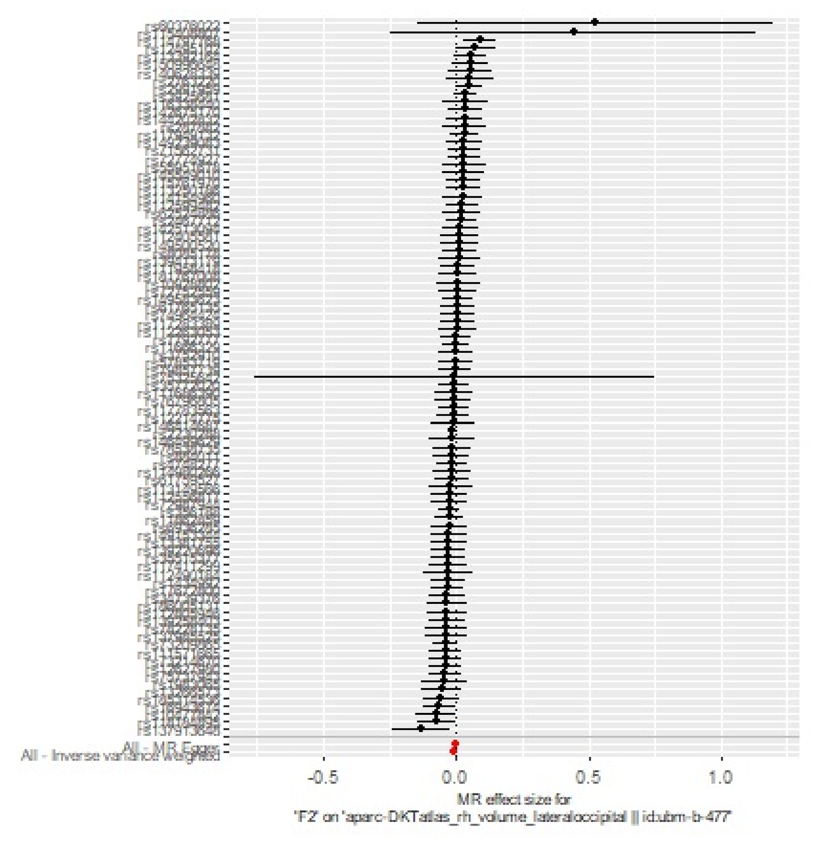

Comparison of results using different MR methods

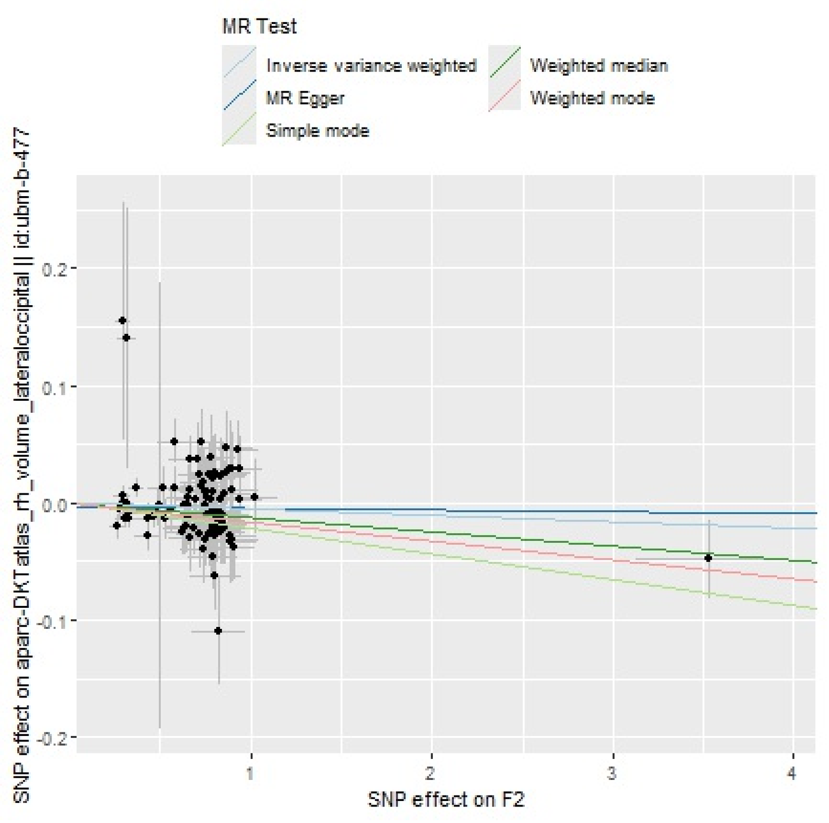

Funnel plot

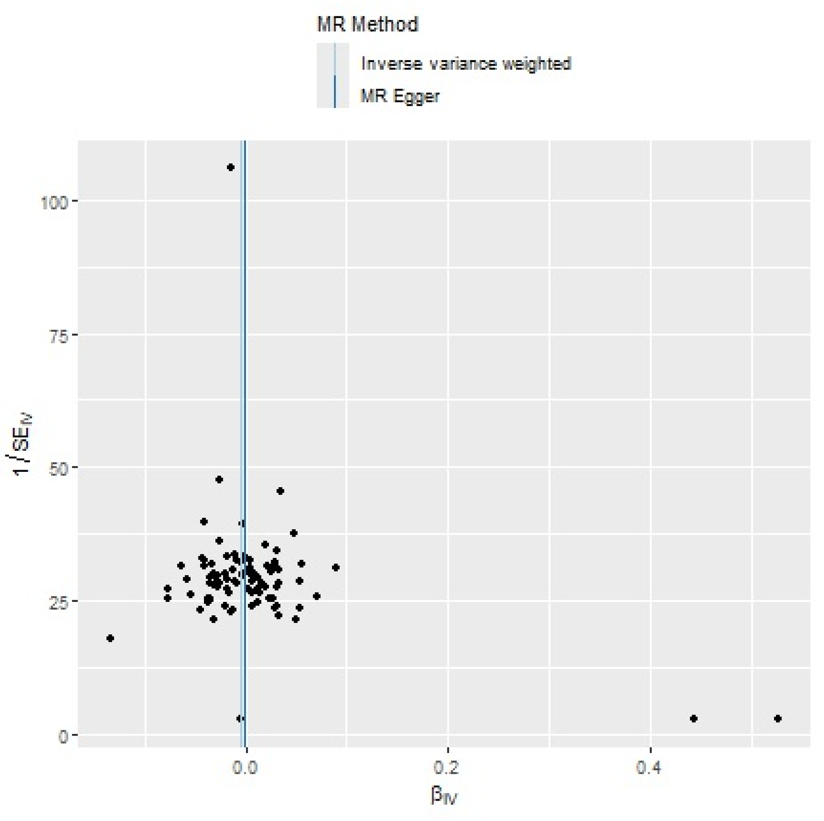

Leave-one-out sensitivity analysis

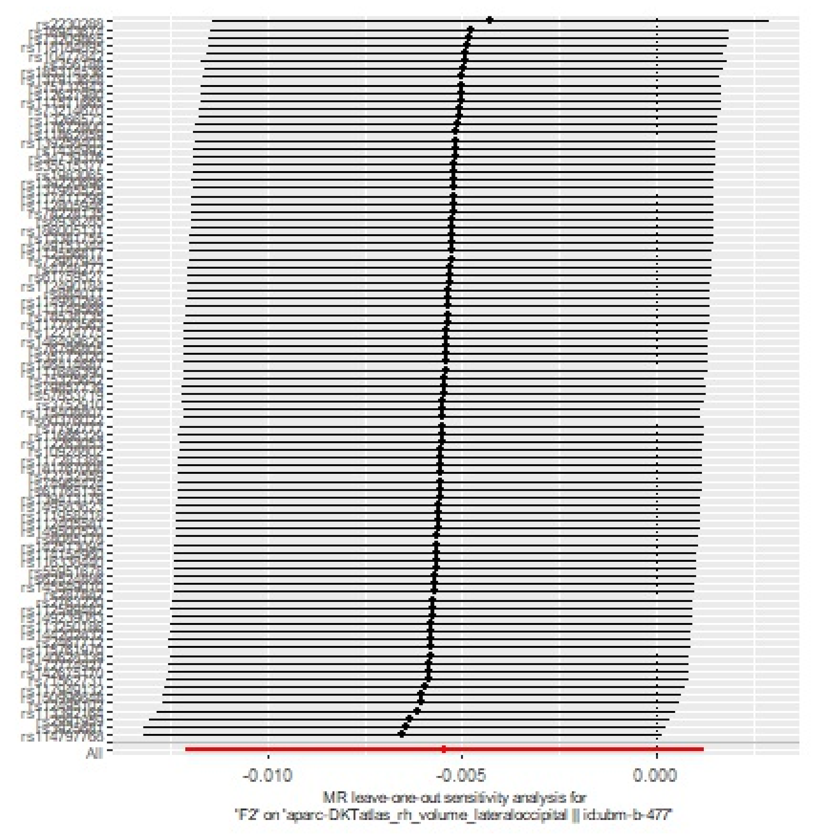

Supplement: Supplementary file 1 [file ijms-26-03578-s001.zip › ijms-3562618-supplementary/TwoSampleMR.F2_against_aparcDKTatlasrhvolumelateraloccipital__idubmb477_SF15.pdf]
