# Supplementary material for: GWAS by Subtraction to Disentangle RBD Genetic Background from α-Synucleinopathies
Source: Int J Mol Sci. 2025 Apr 10;26(8):3578. doi: 10.3390/ijms26083578 (PMC12026788; doi:10.3390/ijms26083578)

Two sample MR report

Two sample MR report

F2 against aseg_global_volume_CC-Anterior || id:ubm-b-186

Date: **10 febbraio, 2025**

Results from two sample MR:

method	nsnp	b	se	pval
MR Egger	91	0.0132080	0.0079287	0.0992619
Weighted median	91	0.0125700	0.0060710	0.0384048
Inverse variance weighted	91	0.0084255	0.0037913	0.0262594
Simple mode	91	0.0176253	0.0127621	0.1706772
Weighted mode	91	0.0132484	0.0080945	0.1051848

Heterogeneity tests

method	Q	Q_df	Q_pval
MR Egger	112.3436	89	0.0479034
Inverse variance weighted	112.9401	90	0.0513710

Test for directional horizontal pleiotropy

egger_intercept	se	pval
-0.0031958	0.0046492	0.4936267

Test that the exposure is upstream of the outcome

snp_r2.exposure	snp_r2.outcome	correct_causal_direction	steiger_pval
0.00605	0.0037266	TRUE	0.1364153

Note - R^2 values are approximate

Forest plot of single SNP MR

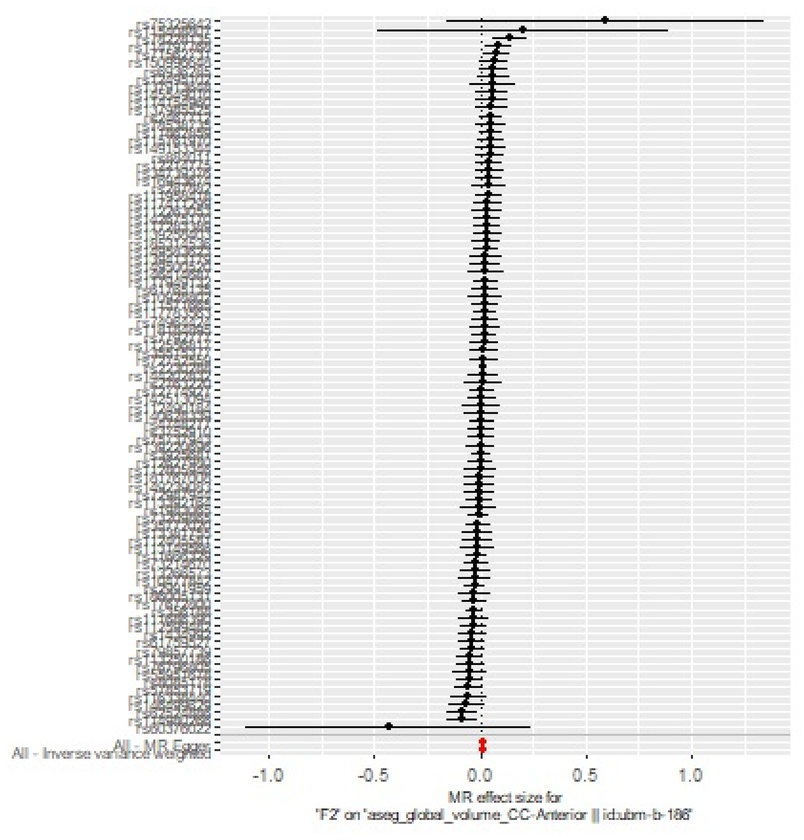

Comparison of results using different MR methods

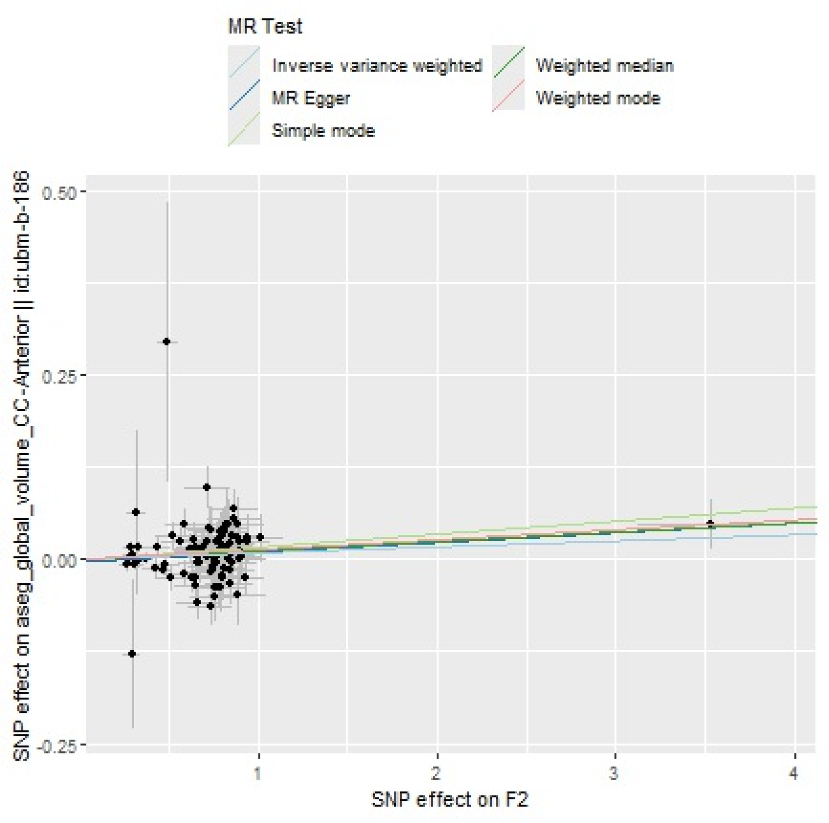

Funnel plot

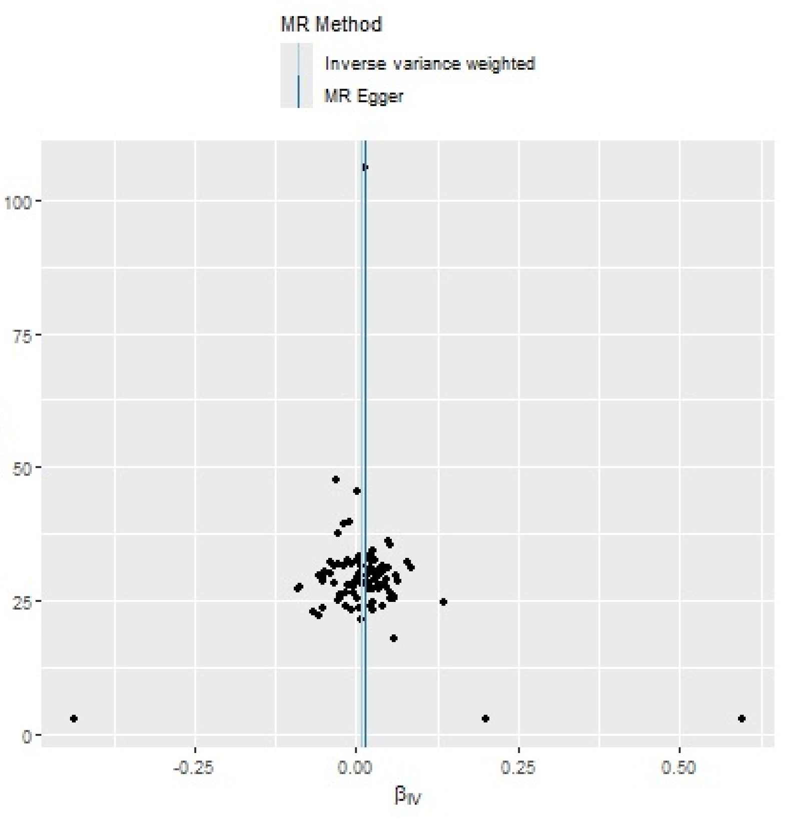

Leave-one-out sensitivity analysis

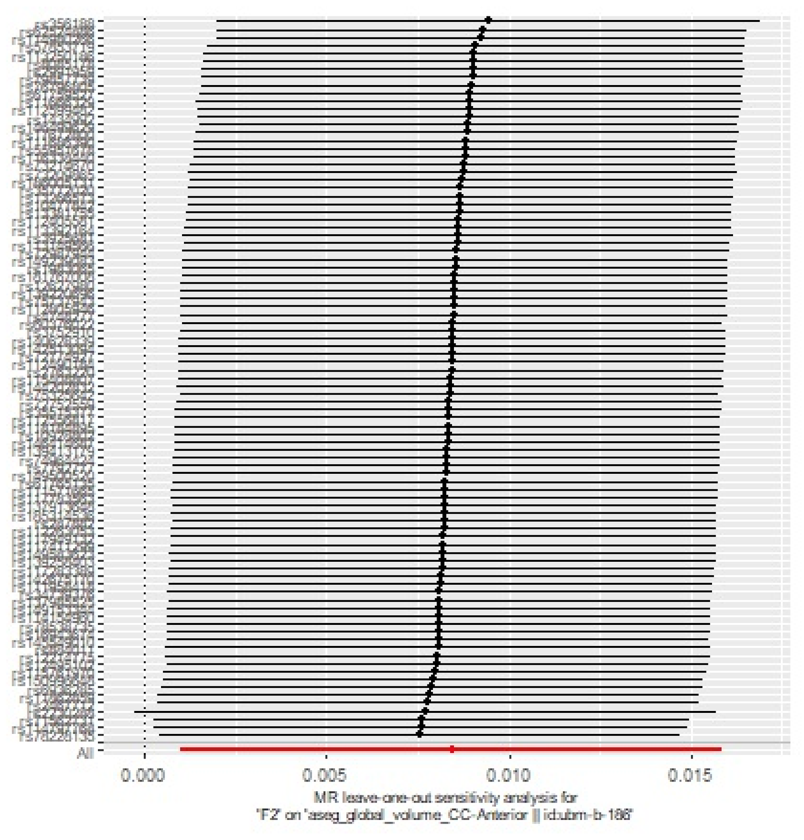

Supplement: Supplementary file 1 [file ijms-26-03578-s001.zip › ijms-3562618-supplementary/TwoSampleMR.F2_against_asegglobalvolumeCCAnterior__idubmb186_SF3.pdf]
