# Supplementary material for: GWAS by Subtraction to Disentangle RBD Genetic Background from α-Synucleinopathies
Source: Int J Mol Sci. 2025 Apr 10;26(8):3578. doi: 10.3390/ijms26083578 (PMC12026788; doi:10.3390/ijms26083578)

Two sample MR report

Two sample MR report

F2 against aseg_global_volume_CC-Mid-Posterior || id:ubm-b-183

Date: **10 febbraio, 2025**

Results from two sample MR:

method	nsnp	b	se	pval
MR Egger	91	0.0086137	0.0070570	0.2254661
Weighted median	91	0.0118107	0.0055762	0.0341711
Inverse variance weighted	91	0.0067270	0.0033844	0.0468490
Simple mode	91	-0.0042248	0.0122579	0.7311547
Weighted mode	91	0.0161536	0.0078272	0.0419196

Heterogeneity tests

method	Q	Q_df	Q_pval
MR Egger	84.58519	89	0.6126891
Inverse variance weighted	84.67801	90	0.6386348

Test for directional horizontal pleiotropy

egger_intercept	se	pval
-0.0012608	0.0041381	0.7613275

Test that the exposure is upstream of the outcome

snp_r2.exposure	snp_r2.outcome	correct_causal_direction	steiger_pval
0.00605	0.0027723	TRUE	0.0254208

Note - R^2 values are approximate

Forest plot of single SNP MR

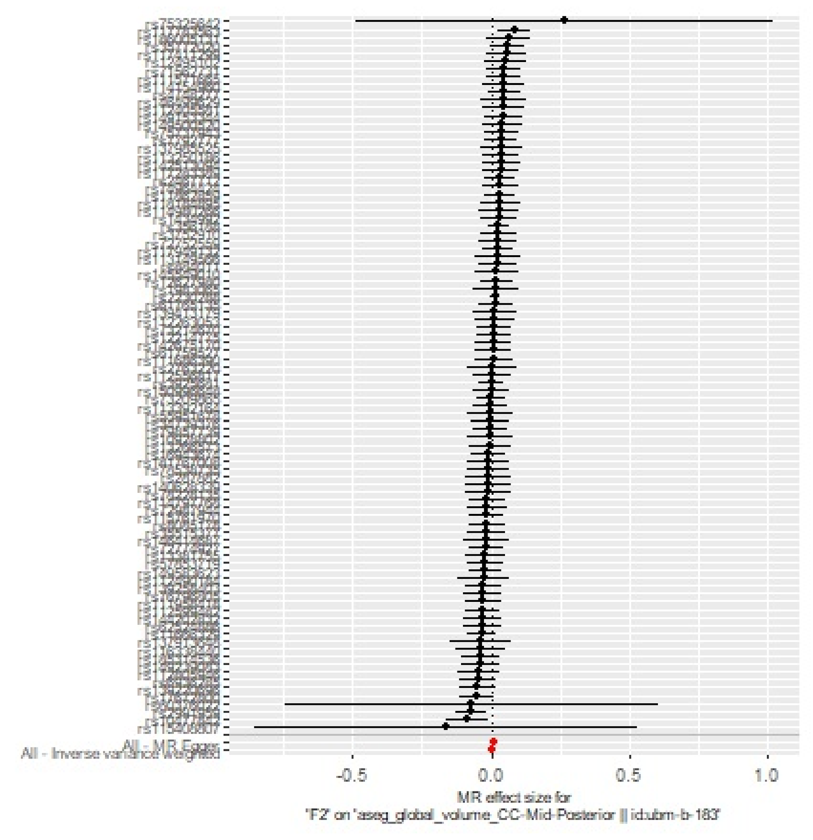

Comparison of results using different MR methods

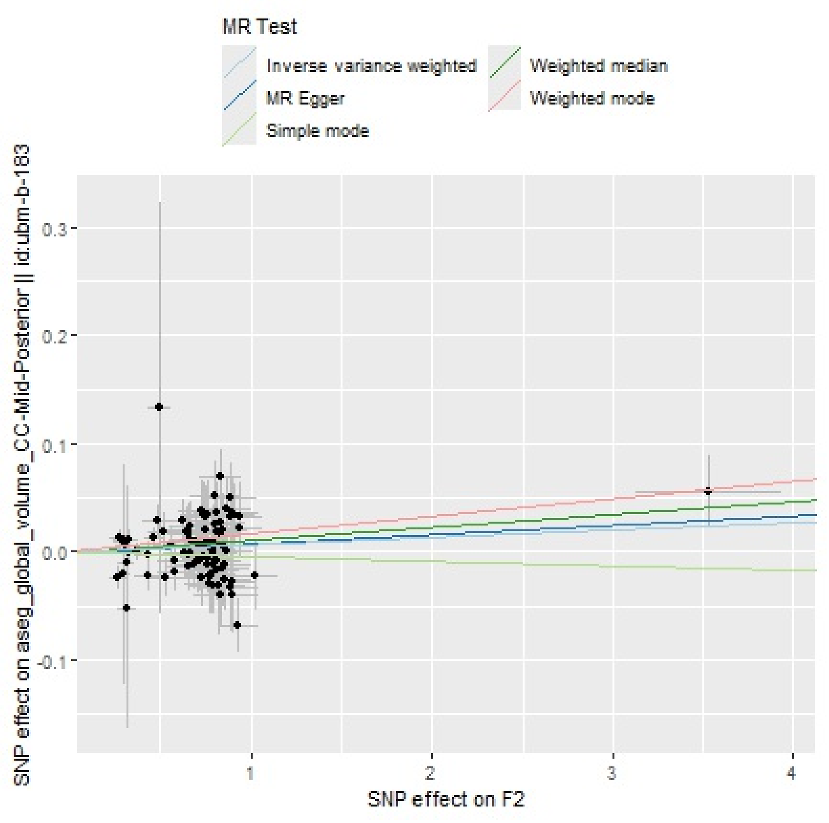

Funnel plot

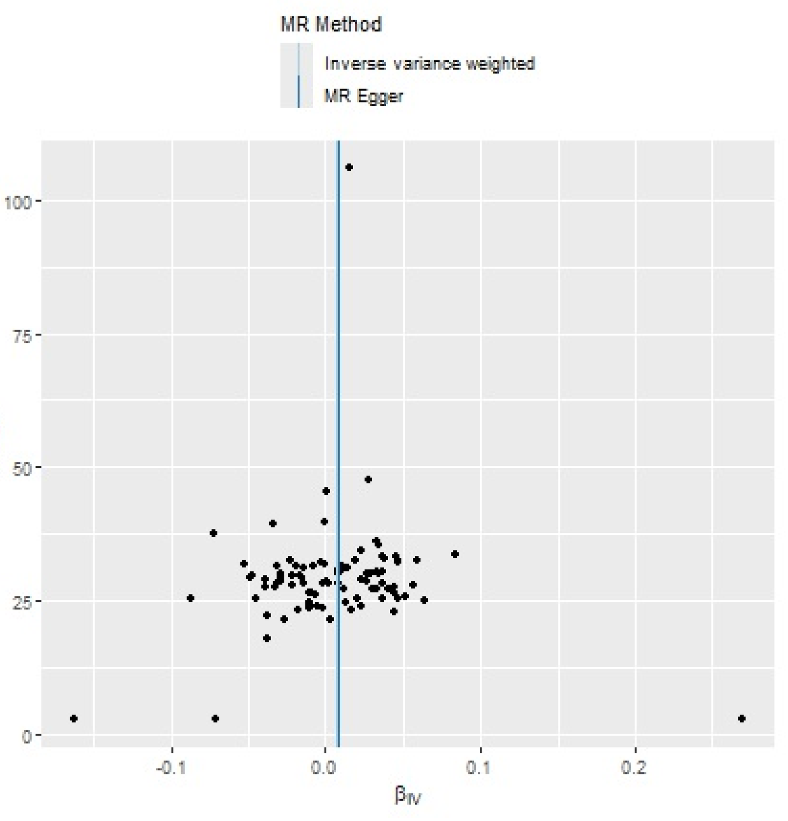

Leave-one-out sensitivity analysis

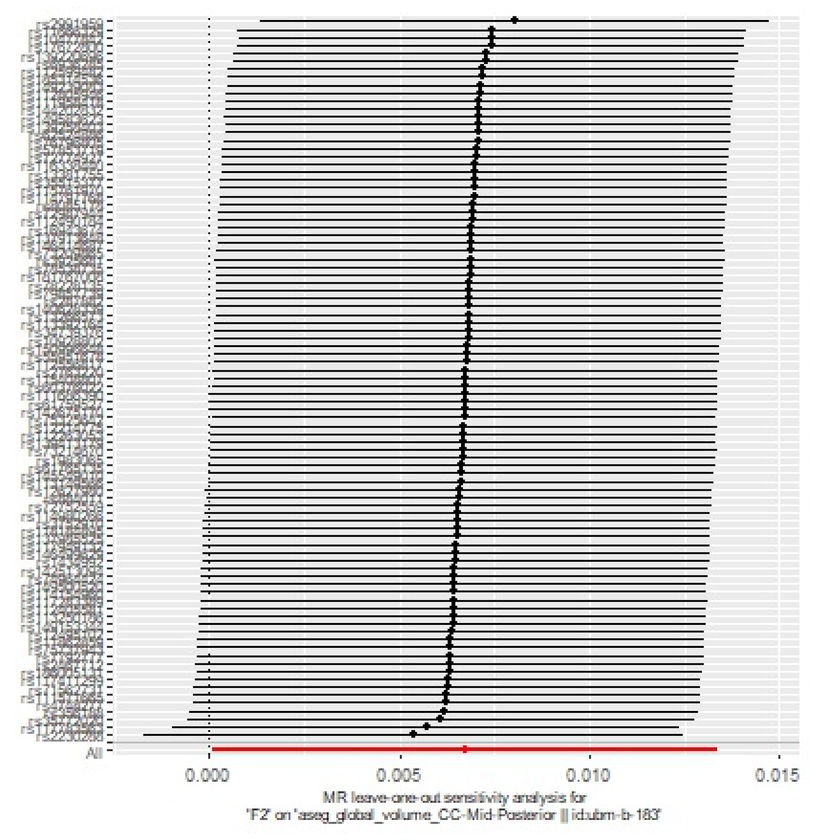

Supplement: Supplementary file 1 [file ijms-26-03578-s001.zip › ijms-3562618-supplementary/TwoSampleMR.F2_against_asegglobalvolumeCCMidPosterior__idubmb183_SF4.pdf]
