# Supplementary material for: GWAS by Subtraction to Disentangle RBD Genetic Background from α-Synucleinopathies
Source: Int J Mol Sci. 2025 Apr 10;26(8):3578. doi: 10.3390/ijms26083578 (PMC12026788; doi:10.3390/ijms26083578)

Two sample MR report

Two sample MR report

F2 against aseg_global_volume_CC-Posterior || id:ubm-b-182

Date: **10 febbraio, 2025**

Results from two sample MR:

method	nsnp	b	se	pval
MR Egger	91	0.0051660	0.0070572	0.4660744
Weighted median	91	0.0119972	0.0060283	0.0465732
Inverse variance weighted	91	0.0069066	0.0033844	0.0412788
Simple mode	91	0.0170997	0.0115091	0.1408393
Weighted mode	91	0.0150952	0.0086729	0.0851899

Heterogeneity tests

method	Q	Q_df	Q_pval
MR Egger	78.95791	89	0.7680798
Inverse variance weighted	79.03691	90	0.7889137

Test for directional horizontal pleiotropy

egger_intercept	se	pval
0.0011631	0.0041382	0.7793071

Test that the exposure is upstream of the outcome

snp_r2.exposure	snp_r2.outcome	correct_causal_direction	steiger_pval
0.00605	0.0026026	TRUE	0.0172959

Note - R^2 values are approximate

Forest plot of single SNP MR

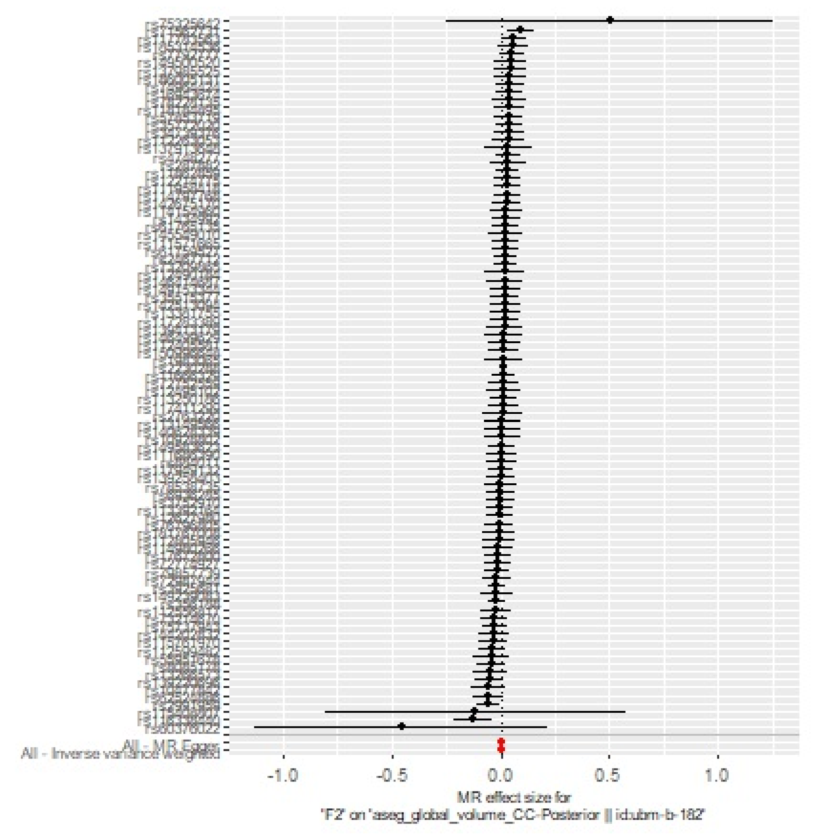

Comparison of results using different MR methods

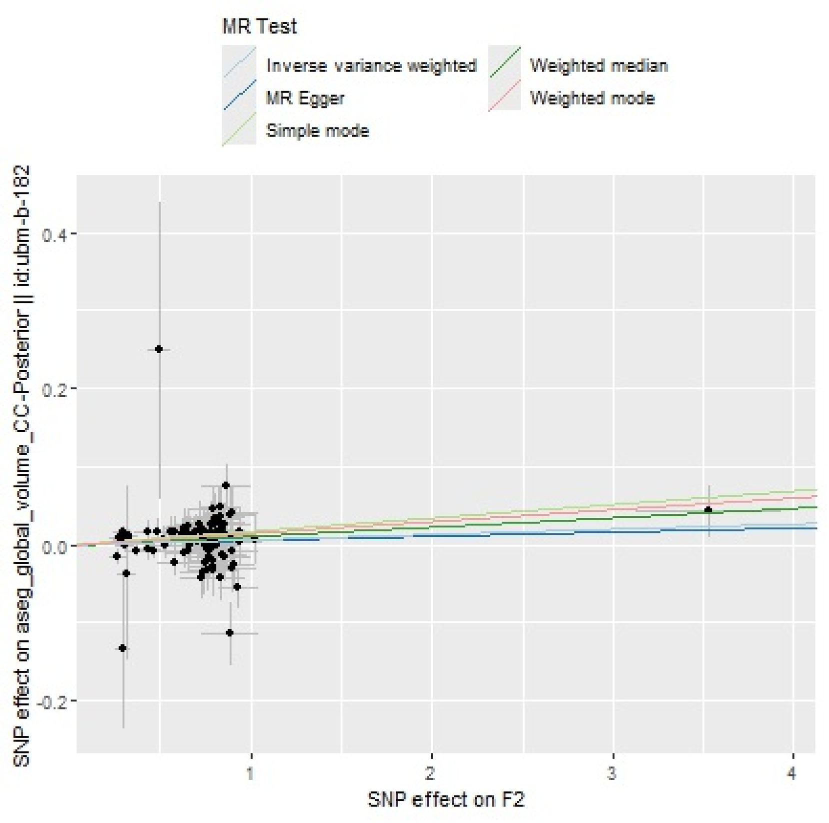

Funnel plot

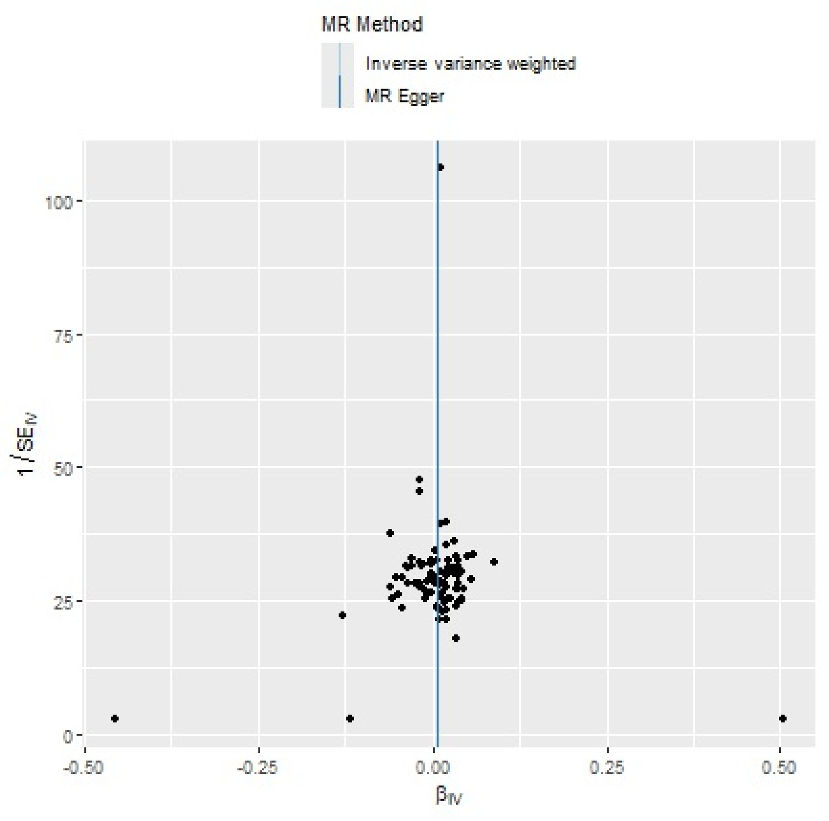

Leave-one-out sensitivity analysis

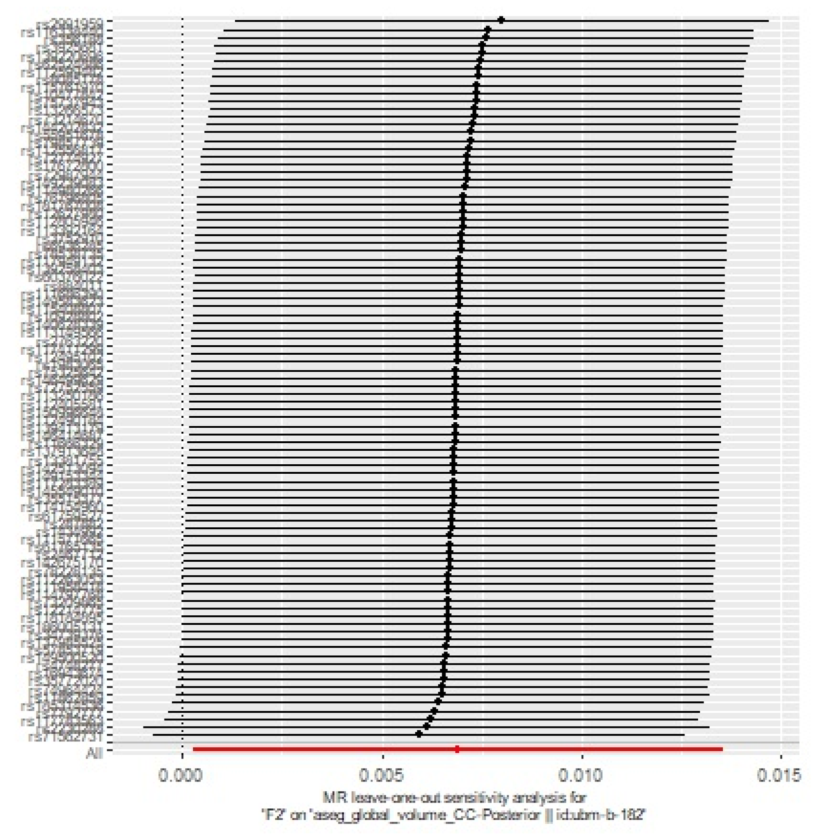

Supplement: Supplementary file 1 [file ijms-26-03578-s001.zip › ijms-3562618-supplementary/TwoSampleMR.F2_against_asegglobalvolumeCCPosterior__idubmb182_SF5.pdf]
