# Supplementary material for: GWAS by Subtraction to Disentangle RBD Genetic Background from α-Synucleinopathies
Source: Int J Mol Sci. 2025 Apr 10;26(8):3578. doi: 10.3390/ijms26083578 (PMC12026788; doi:10.3390/ijms26083578)

Two sample MR report

Two sample MR report

F2 against aseg_lh_volume_Cerebellum-Cortex || id:ubm-b-194

Date: **10 febbraio, 2025**

Results from two sample MR:

method	nsnp	b	se	pval
MR Egger	91	0.0267923	0.0072301	0.0003657
Weighted median	91	0.0072250	0.0059731	0.2264301
Inverse variance weighted	91	0.0042002	0.0036855	0.2544259
Simple mode	91	0.0009441	0.0129473	0.9420296
Weighted mode	91	0.0061356	0.0082547	0.4592424

Heterogeneity tests

method	Q	Q_df	Q_pval
MR Egger	93.41665	89	0.3536399
Inverse variance weighted	106.72607	90	0.1101236

Test for directional horizontal pleiotropy

egger_intercept	se	pval
-0.0150967	0.0042396	0.0005959

Test that the exposure is upstream of the outcome

snp_r2.exposure	snp_r2.outcome	correct_causal_direction	steiger_pval
0.00605	0.0033865	TRUE	0.0813791

Note - R^2 values are approximate

Forest plot of single SNP MR

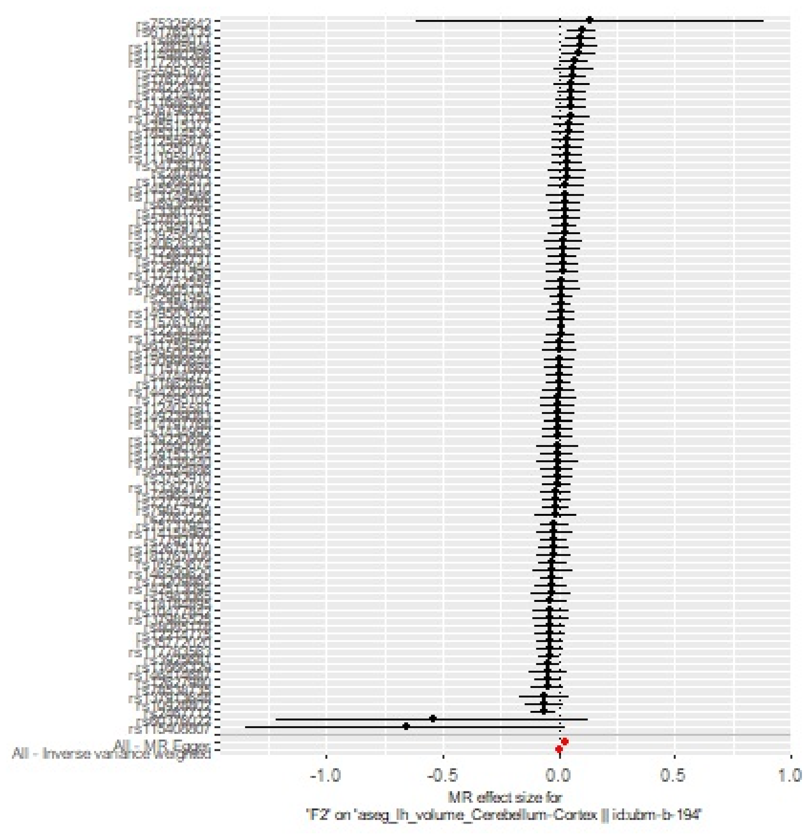

Comparison of results using different MR methods

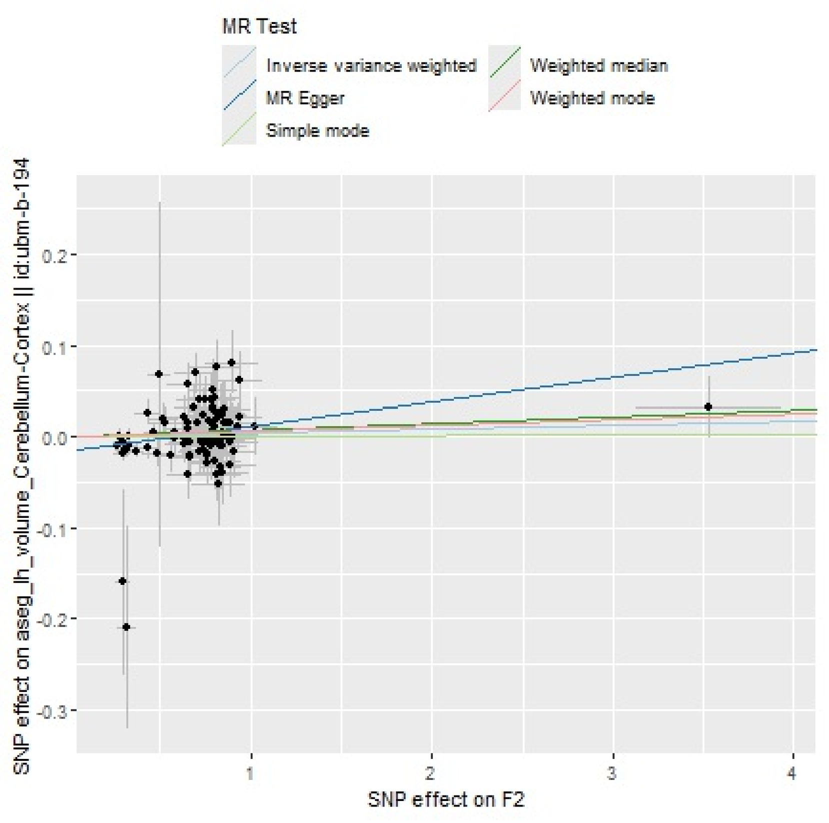

Funnel plot

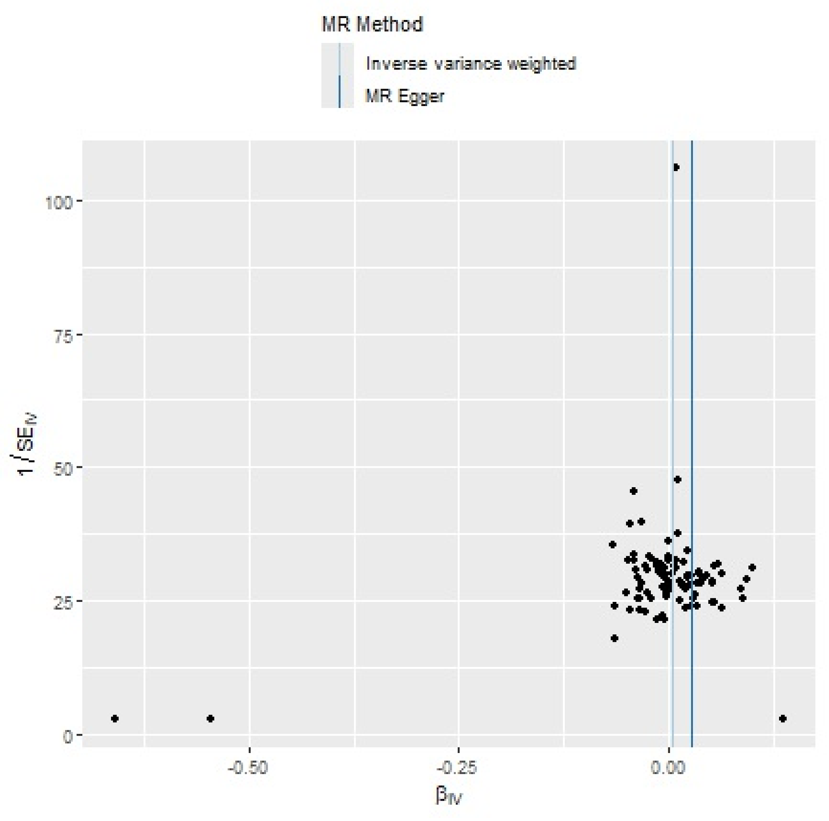

Leave-one-out sensitivity analysis

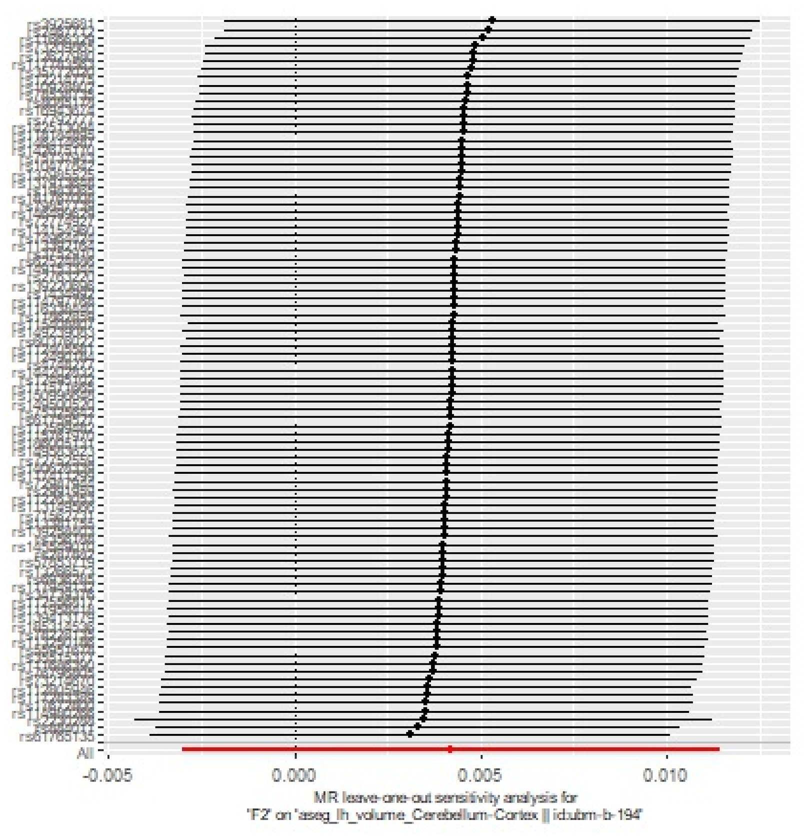

Supplement: Supplementary file 1 [file ijms-26-03578-s001.zip › ijms-3562618-supplementary/TwoSampleMR.F2_against_aseglhvolumeCerebellumCortex__idubmb194_SF8.pdf]
