# Supplementary material for: GWAS by Subtraction to Disentangle RBD Genetic Background from α-Synucleinopathies
Source: Int J Mol Sci. 2025 Apr 10;26(8):3578. doi: 10.3390/ijms26083578 (PMC12026788; doi:10.3390/ijms26083578)

Two sample MR report

Two sample MR report

F2 against aseg_lh_volume_Cerebellum-White-Matter || id:ubm-b-193

Date: **10 febbraio, 2025**

Results from two sample MR:

method	nsnp	b	se	pval
MR Egger	91	0.0184893	0.0070931	0.0107188
Weighted median	91	0.0050218	0.0061864	0.4169301
Inverse variance weighted	91	0.0064461	0.0034531	0.0619372
Simple mode	91	0.0008836	0.0114334	0.9385696
Weighted mode	91	0.0028589	0.0073973	0.7000523

Heterogeneity tests

method	Q	Q_df	Q_pval
MR Egger	89.90810	89	0.4531178
Inverse variance weighted	93.69013	90	0.3741339

Test for directional horizontal pleiotropy

egger_intercept	se	pval
-0.0080477	0.0041592	0.0561784

Test that the exposure is upstream of the outcome

snp_r2.exposure	snp_r2.outcome	correct_causal_direction	steiger_pval
0.00605	0.0030441	TRUE	0.0443183

Note - R^2 values are approximate

Forest plot of single SNP MR

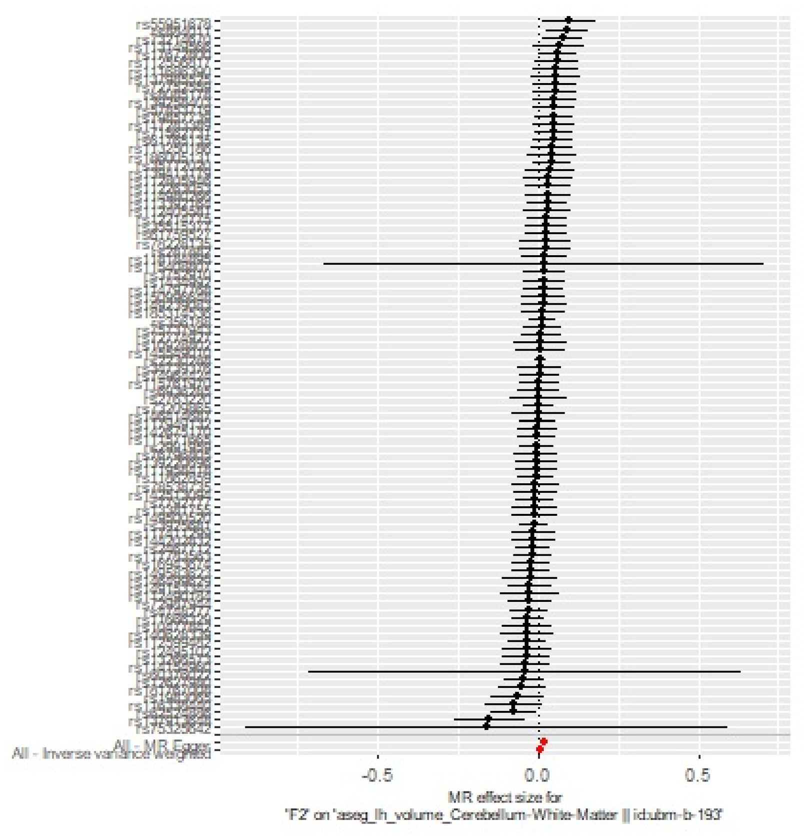

Comparison of results using different MR methods

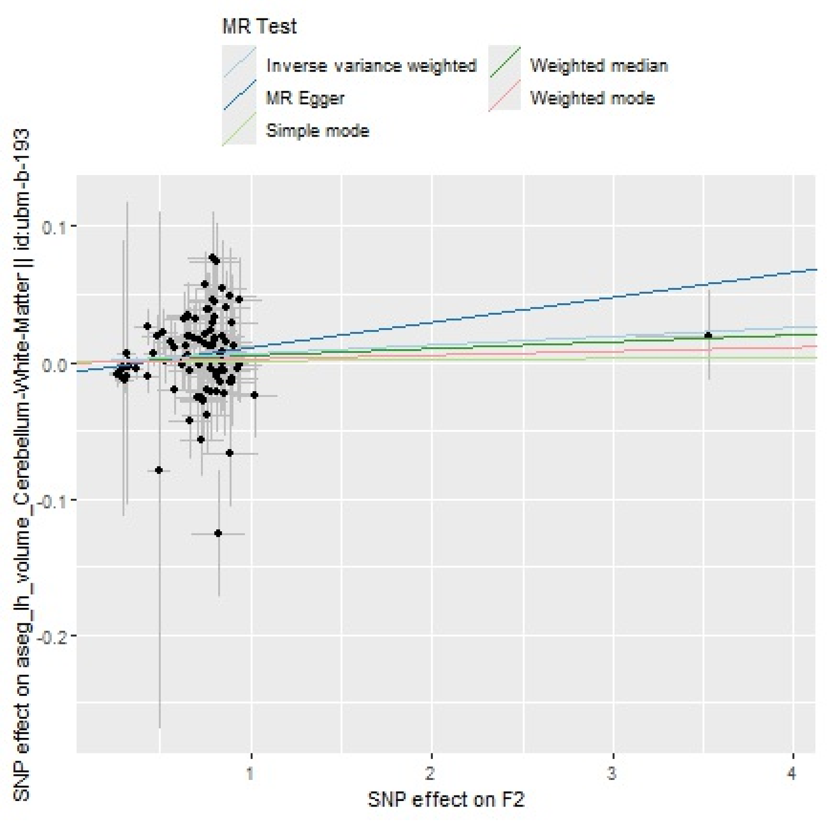

Funnel plot

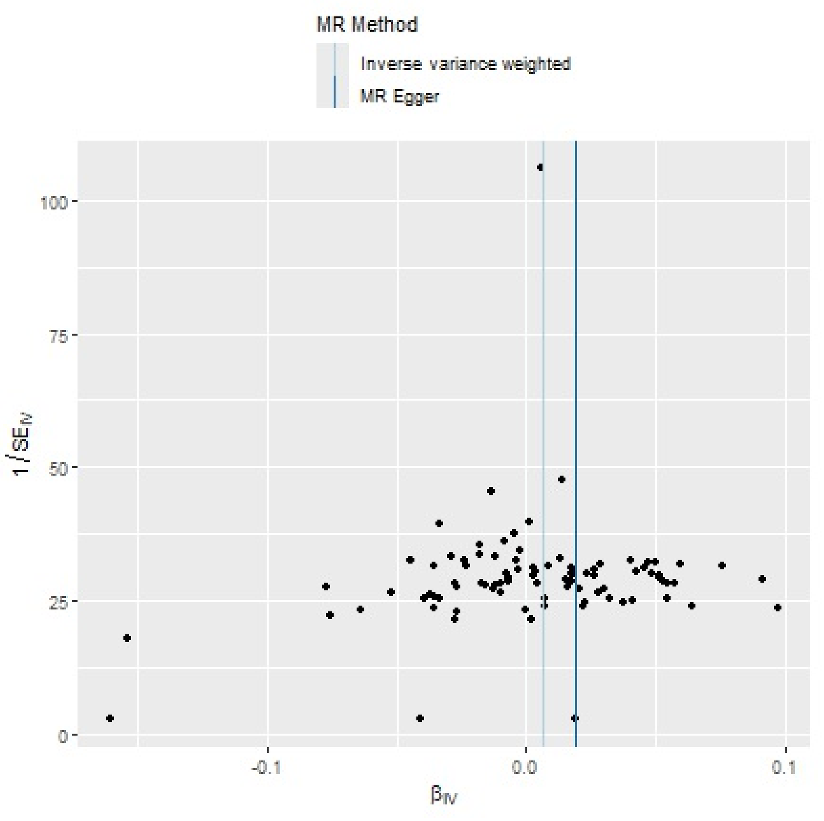

Leave-one-out sensitivity analysis

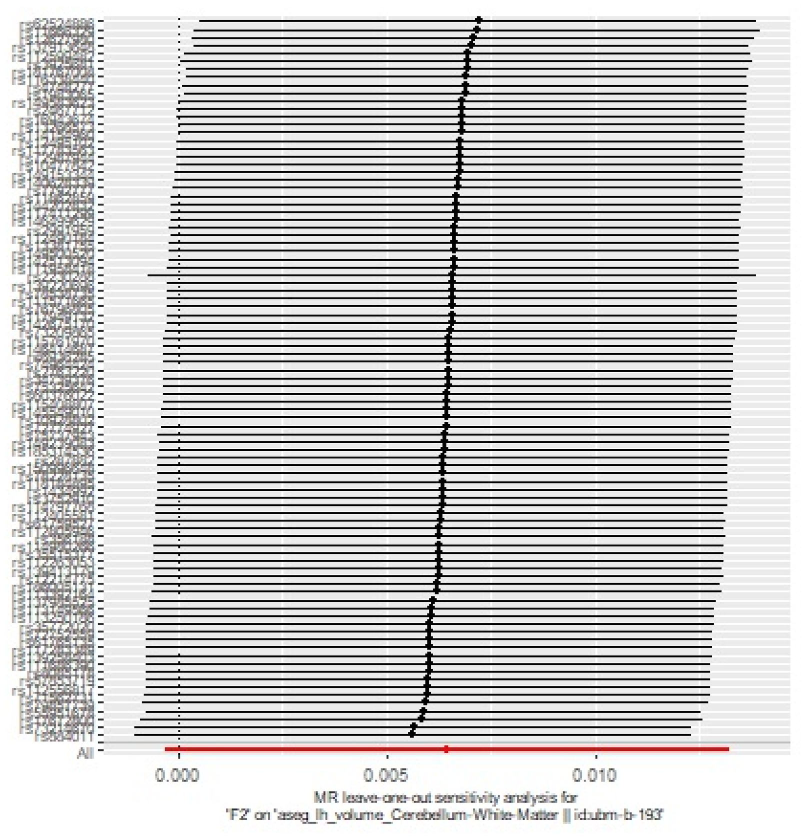

Supplement: Supplementary file 1 [file ijms-26-03578-s001.zip › ijms-3562618-supplementary/TwoSampleMR.F2_against_aseglhvolumeCerebellumWhiteMatter__idubmb193_SF10.pdf]
