# Supplementary material for: GWAS by Subtraction to Disentangle RBD Genetic Background from α-Synucleinopathies
Source: Int J Mol Sci. 2025 Apr 10;26(8):3578. doi: 10.3390/ijms26083578 (PMC12026788; doi:10.3390/ijms26083578)

Two sample MR report

Two sample MR report

F2 against aseg_rh_volume_Cerebellum-Cortex || id:ubm-b-211

Date: **10 febbraio, 2025**

Results from two sample MR:

method	nsnp	b	se	pval
MR Egger	91	0.0216616	0.0073012	0.0038633
Weighted median	91	0.0073569	0.0061430	0.2310724
Inverse variance weighted	91	0.0064798	0.0035901	0.0710884
Simple mode	91	-0.0165980	0.0137152	0.2293736
Weighted mode	91	0.0077697	0.0086793	0.3730660

Heterogeneity tests

method	Q	Q_df	Q_pval
MR Egger	95.26262	89	0.3055396
Inverse variance weighted	101.27282	90	0.1957988

Test for directional horizontal pleiotropy

egger_intercept	se	pval
-0.010145	0.0042813	0.0199676

Test that the exposure is upstream of the outcome

snp_r2.exposure	snp_r2.outcome	correct_causal_direction	steiger_pval
0.00605	0.0032825	TRUE	0.0683391

Note - R^2 values are approximate

Forest plot of single SNP MR

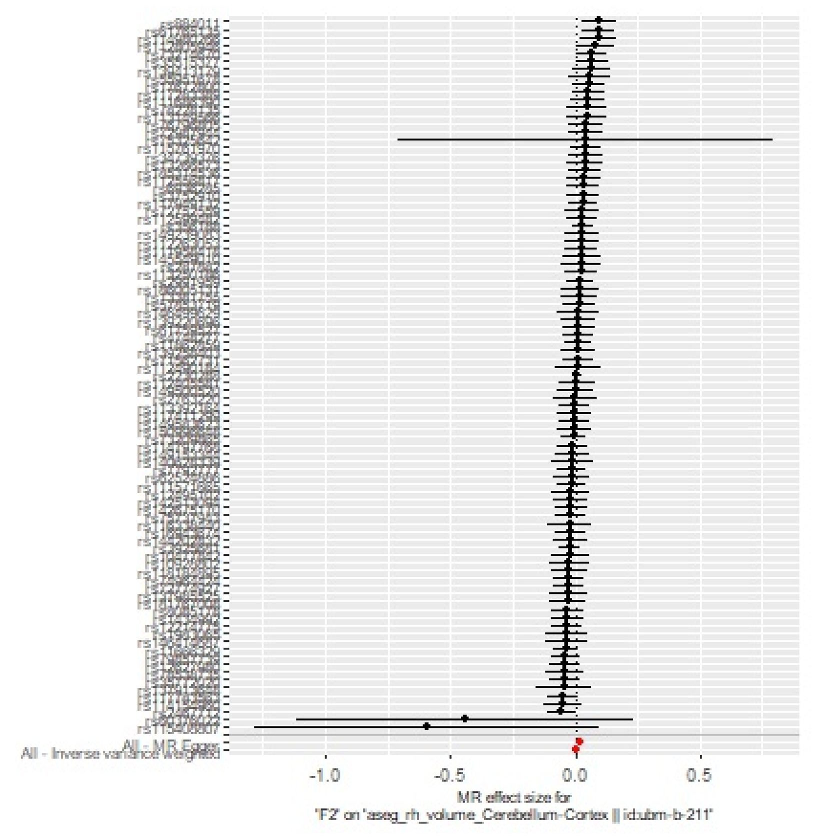

Comparison of results using different MR methods

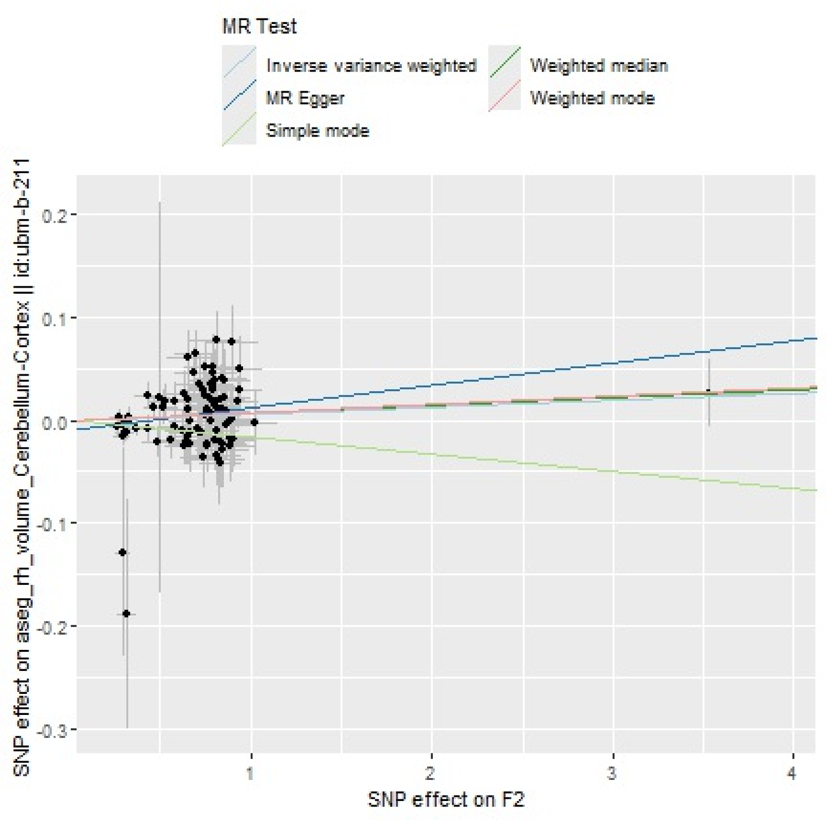

Funnel plot

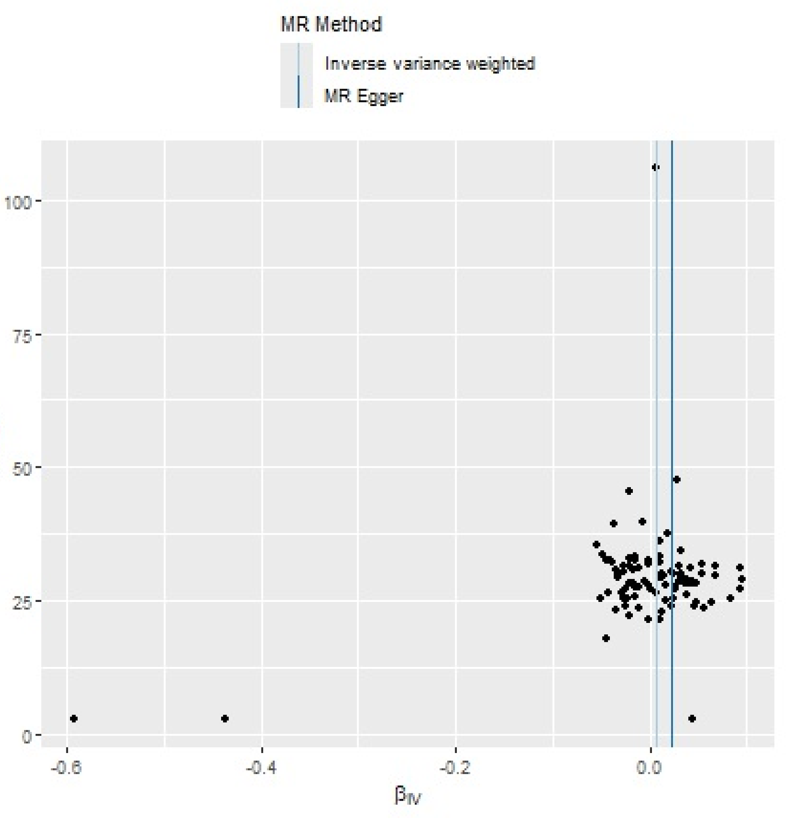

Leave-one-out sensitivity analysis

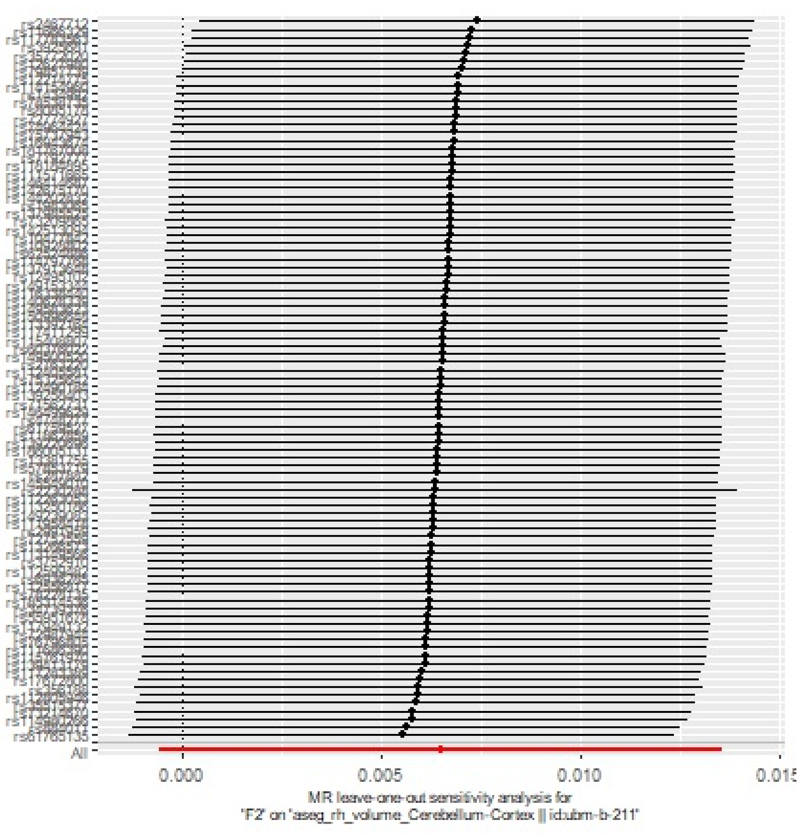

Supplement: Supplementary file 1 [file ijms-26-03578-s001.zip › ijms-3562618-supplementary/TwoSampleMR.F2_against_asegrhvolumeCerebellumCortex__idubmb211_SF9.pdf]
