# Supplementary material for: GWAS by Subtraction to Disentangle RBD Genetic Background from α-Synucleinopathies
Source: Int J Mol Sci. 2025 Apr 10;26(8):3578. doi: 10.3390/ijms26083578 (PMC12026788; doi:10.3390/ijms26083578)

Two sample MR report

Two sample MR report

F2 against aseg_rh_volume_Cerebellum-White-Matter || id:ubm-b-210

Date: **10 febbraio, 2025**

Results from two sample MR:

method	nsnp	b	se	pval
MR Egger	91	0.0270862	0.0070569	0.0002314
Weighted median	91	0.0075695	0.0052605	0.1501709
Inverse variance weighted	91	0.0083920	0.0034927	0.0162752
Simple mode	91	0.0011925	0.0121333	0.9219241
Weighted mode	91	0.0118306	0.0085249	0.1686352

Heterogeneity tests

method	Q	Q_df	Q_pval
MR Egger	86.74239	89	0.5479826
Inverse variance weighted	95.85616	90	0.3167445

Test for directional horizontal pleiotropy

egger_intercept	se	pval
-0.0124924	0.0041381	0.0033094

Test that the exposure is upstream of the outcome

snp_r2.exposure	snp_r2.outcome	correct_causal_direction	steiger_pval
0.00605	0.0031907	TRUE	0.0581637

Note - R^2 values are approximate

Forest plot of single SNP MR

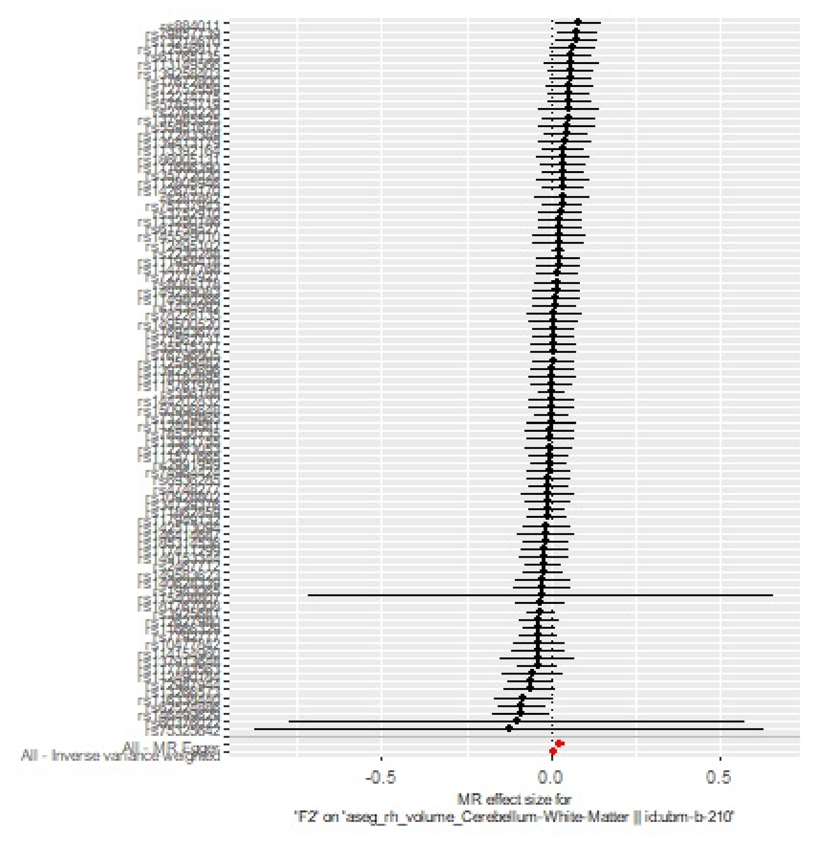

Comparison of results using different MR methods

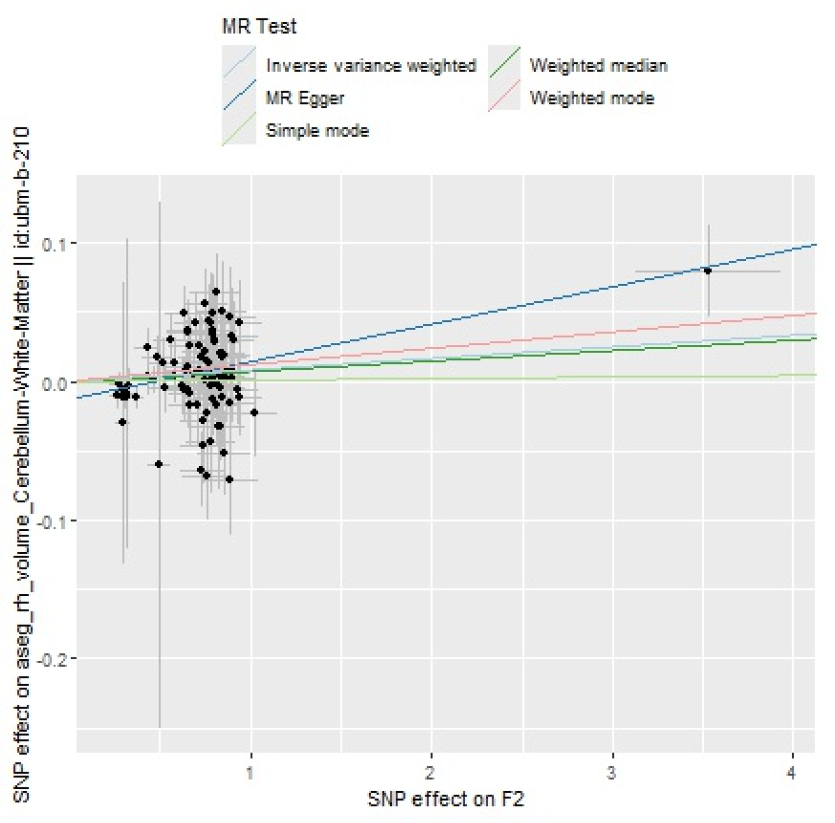

Funnel plot

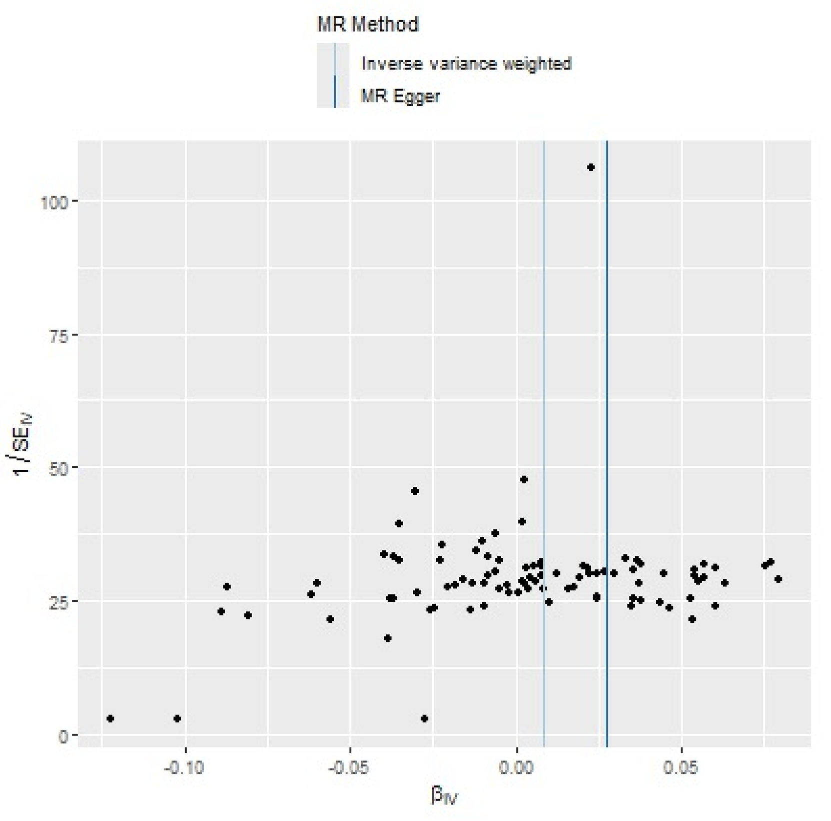

Leave-one-out sensitivity analysis

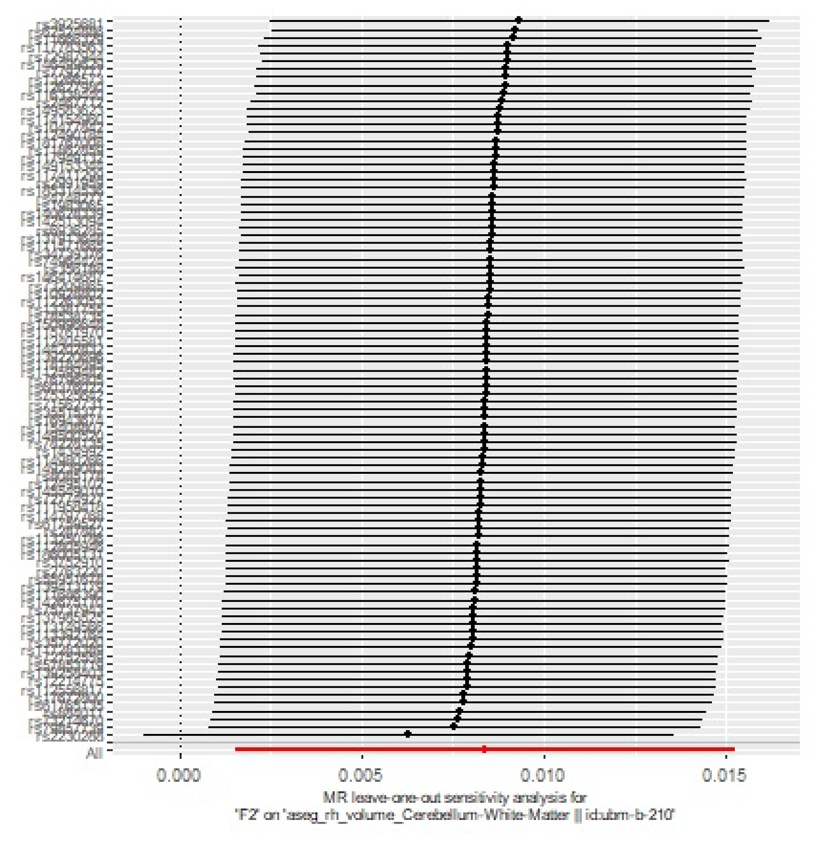

Supplement: Supplementary file 1 [file ijms-26-03578-s001.zip › ijms-3562618-supplementary/TwoSampleMR.F2_against_asegrhvolumeCerebellumWhiteMatter__idubmb210_SF11.pdf]
